# Systems metabolic engineering of *Corynebacterium glutamicum* for production of the chemical chaperone ectoine

**DOI:** 10.1186/1475-2859-12-110

**Published:** 2013-11-15

**Authors:** Judith Becker, Rudolf Schäfer, Michael Kohlstedt, Björn J Harder, Nicole S Borchert, Nadine Stöveken, Erhard Bremer, Christoph Wittmann

**Affiliations:** 1 Institute of Biochemical Engineering, Technische Universität Braunschweig, Braunschweig, Germany; 2 Department of Biology, Laboratory for Microbiology, Philipps-University Marburg, Marburg, Germany; 3 LOEWE-Center for Synthetic Microbiology, Philipps-University Marburg, Marburg, Germany

**Keywords:** Synthetic biology, Metabolic engineering, Compatible solutes, Chemical chaperones, Heterologous production, Aspartokinase, Codon optimization

## Abstract

**Background:**

The stabilizing and function-preserving effects of ectoines have attracted considerable biotechnological interest up to industrial scale processes for their production. These rely on the release of ectoines from high-salinity-cultivated microbial producer cells upon an osmotic down-shock in rather complex processor configurations. There is growing interest in uncoupling the production of ectoines from the typical conditions required for their synthesis, and instead design strains that naturally release ectoines into the medium without the need for osmotic changes, since the use of high-salinity media in the fermentation process imposes notable constraints on the costs, design, and durability of fermenter systems.

**Results:**

Here, we used a *Corynebacterium glutamicum* strain as a cellular chassis to establish a microbial cell factory for the biotechnological production of ectoines. The implementation of a mutant aspartokinase enzyme ensured efficient supply of L-aspartate-beta-semialdehyde, the precursor for ectoine biosynthesis. We further engineered the genome of the basic *C. glutamicum* strain by integrating a codon-optimized synthetic *ectABCD* gene cluster under expressional control of the strong and constitutive *C. glutamicum tuf* promoter. The resulting recombinant strain produced ectoine and excreted it into the medium; however, lysine was still found as a by-product. Subsequent inactivation of the L-lysine exporter prevented the undesired excretion of lysine while ectoine was still exported. Using the streamlined cell factory, a fed-batch process was established that allowed the production of ectoine with an overall productivity of 6.7 g L^-1^ day^-1^ under growth conditions that did not rely on the use of high-salinity media.

**Conclusions:**

The present study describes the construction of a stable microbial cell factory for recombinant production of ectoine. We successfully applied metabolic engineering strategies to optimize its synthetic production in the industrial workhorse *C. glutamicum* and thereby paved the way for further improvements in ectoine yield and biotechnological process optimization.

## Background

**Figure 1:**
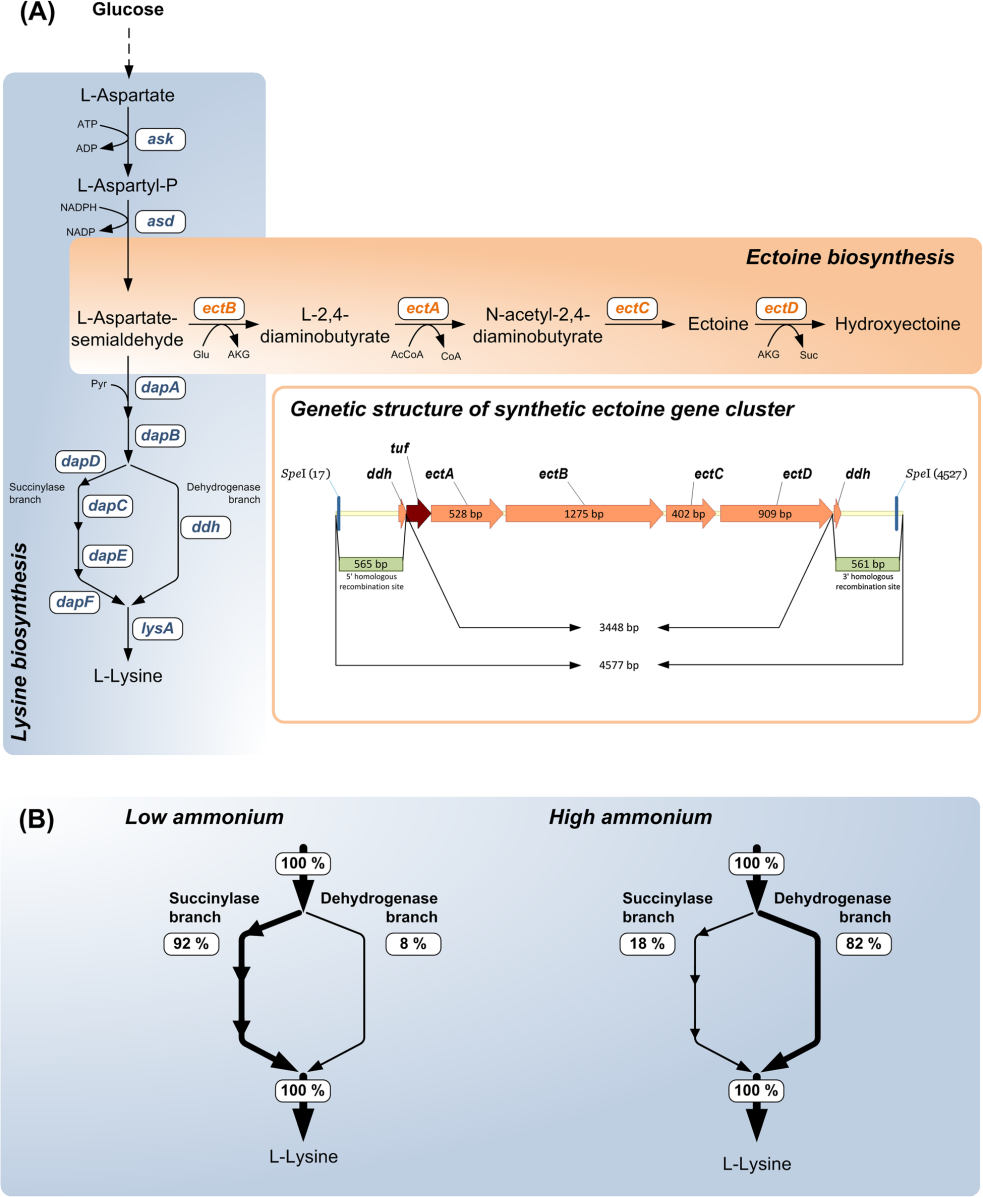
**Metabolic engineering strategy for heterologous production of ectoine and 5**-**hydroxyectoine in *Corynebacterium glutamicum *from the building block L**-**aspartate-β-semialdehyde.** L-aspartate-β-semialdehyde is synthesized through the concerted actions of the aspartokinase (Ask; EC: 2.7.2.4) and aspartate-semialdehyde-dehydrogenase (Asd; EC: 1.2.1.11). It is then converted into the compatible solutes ectoine and 5-hydroxyectoine, respectively, by the L-2,4-diaminobutyrate transaminase (EctB; EC: 2.6.1.76) to form L-2,4-diaminobutyrate, a metabolite that is then acetylated by the 2,4-diaminobutyrate acetyltransferase (EctA; EC: 2.3.1.178) to produce *N*-γ-acetyl-2,4-diaminobutyrate, which is subsequently cyclized via a water elimination reaction by the ectoine synthase (EctC; EC: 4.2.1.108), to yield ectoine. Ectoine can then serve as the substrate for the formation of 5-hydroxyectoine through the activity of ectoine hydroxylase (EctD; EC: 1.14.11). Heterologous production in *C. glutamicum* was mediated via the codon-optimized *ectABCD* gene cluster based on that present in *P. stutzeri* A1501. The synthetic gene cluster was designed to be constitutively expressed from the promoter for the *tuf* gene from *C. glutamicum*. For genome-based integration via double-recombination event, the construct was equipped with flanking regions of about 560 bp DNA sequences derived from the upstream and downstream regions of the *ddh* gene. Recognition sites for the restriction enzyme *Spe*I were added to facilitate cloning of this DNA fragment into the vector pClik_int_*sacB***(A)**. The *ddh* gene, encoding diaminopimelate dehydrogenase, was chosen as integration site to minimize competing carbon flux towards lysine. Tracer studies with 3-^13^C glucose identified this biosynthetic branch as major contributor to the overall lysine flux under conditions with high ammonium availability which is readily present under industrial-scale production conditions **(B)**.

Many microorganisms counteract the detrimental effects of high salinity and high osmolarity through the accumulation of water-attracting organic osmolytes, the so-called compatible solutes [1-3]. The term “chemical chaperone” has been coined in the literature to characterize the cellular functions of these compounds as stabilizers of macromolecules [4-6]. Among them, the tetrahydropyrimidines ectoine and 5-hydroxyectoine have been most widely adopted in practical applications, and their biotechnological production has been advanced to an industrial scale [7,8]. Ectoines possess excellent stabilizing effects on biological molecules; e.g. proteins, cell membranes, DNA, and even entire cells. They safeguard proteins against aggregation, promote their proper folding under otherwise denaturing conditions, and they are fully compliant with cellular physiology, biochemistry and protein functions [9-12]. Related to these attractive properties, industry has merchandized ectoines as protective compounds for health care products and cosmetics [7,13,14]. To some extent they also find application as *in vivo* folding catalyst for the recombinant production of proteins [15] and as enhancers for polymerase chain reactions [16]. The anti-inflammatory effect of ectoine even suggests a medical oriented application in the future for treating lung inflammation [17] and colitis [18], and for tissue protection in ischemia [19]. Although the chemical synthesis of ectoines is certainly possible [16], their large-scale production with a high degree of purity and stereo-specificity is complicated and costly. Chemical synthesis was consequently outcompeted by a biotechnological production route using the halophilic bacterium *Halomonas elongata *[20]. *In vivo*, ectoine is synthesized from the precursor L-aspartate-β-semialdehyde (ASA), a central hub in microbial amino acid production [21], by three successive enzymatic steps that are catalyzed by the L-2,4-diaminobutyrate transaminase (EctB), the 2,4-diaminobutyrate acetyltransferase (EctA) and the ectoine synthase (EctC) [22,23] (Figure 1A). A substantial subgroup of the ectoine producers can convert ectoine into 5-hydroxyectoine through the activity of the ectoine hydroxylase (EctD) [24,25] (Figure 1A). The ectoine biosynthetic genes are normally organized in an operon (*ectABC*) [23,26-28] that might also comprise the *ectD* gene [24,29,30]. Expression is typically induced in response to increased osmolarity [8,26,27,31] or extremes in growth temperature [27,31]. Transcriptional profiling indicated that the cellular levels of ASA could be a potential bottleneck in the synthesis of ectoines [32]. Some natural producers can avert this through the co-production of an aspartokinase (Ask_Ect) with special biochemical features [33].

The industrial ectoine production process - popularly referred to as “*bacterial milking*” - entails the fermentation of *H. elongata* under high-salinity growth conditions and a subsequent rapid osmotic downshift to release the produced ectoine from the cells through the transient opening of mechanosensitive channels [34,35]. While the bacterial milking procedure is an expedient way to recover ectoine under industrial settings [7,8,20], concerted efforts have recently been made to enhance the efficiency and convenience of its production. These efforts include the use of mutants of *H. elongata* that hyper-secrete ectoine [36], alternative microbial production strains [33,37-39], modifications of the fermentation and milking procedures [38-42], and attempts to raise production through recombinant DNA techniques employing both pro- and eukaryotic host systems [33,37,43-45]. In particular, attempts are being made to uncouple ectoine production from high osmolarity since the use of high-salinity media in the fermentation process imposes notable constraints on the costs, design, and durability of fermenter systems.

We considered the non-pathogenic Gram-positive soil bacterium *Corynebacterium glutamicum*, a well-established industrial workhorse [46], as an excellent candidate for the development of a synthetic production platform of ectoines. Owing to the wide use of *C. glutamicum* in biotechnology, in-depth knowledge on its large-scale fermentation as well as genetic tools are available to metabolically direct and streamline the production of commercially interesting metabolites [47-50]. Keeping in mind that the synthesis of ectoine proceeds from ASA (Figure 1A), *C. glutamicum* seemed to be a particular well suitable chassis, as biotechnological manufacturing of L-lysine has bred feedback-resistant aspartokinase enzymes [51]. As *C. glutamicum* is not a natural ectoine/hydroxyectoine producer [52], we designed a synthetic cell factory recruiting the *ectABCD* gene cluster originating from *Pseudomonas stutzeri* A1501. The genes were codon-optimized and expression was uncoupled from its normal osmotic stress-induced transcriptional control by employing a promoter that is constitutively active in *C. glutamicum*. We demonstrate here that the newly constructed recombinant *C. glutamicum* strain excretes most of the formed ectoine/hydroxyectoine into the growth medium. We further optimized its performance by elimination of by-product formation through metabolic engineering and benchmarked the ectoine production performance in fed-batch fermentation.

## Results

### Design of the cellular chassis for ectoine synthesis

For heterologous synthesis of ectoine in *C. glutamicum*, a basic lysine-producer was chosen as a suitable genetic background. This strain, *C. glutamicum* LYS-1, possesses a feedback-resistant aspartokinase (LysC-T311I) [53] and thereby circumvents the native biochemical pathway regulation for the synthesis of ASA that normally keeps the cellular pool of this central metabolite under tight control [54]. Thus, an adequate supply of the precursor molecule for ectoine biosynthesis (Figure 1A) was aimed with the chosen *C. glutamicum* starter strain LYS-1. This strain is known to overproduce lysine which might arise as potential by-product in the aspired ectoine producer. In *C. glutamicum*, lysine can be formed via two alternative metabolic routes – the succinylase pathway and the dehydrogenase pathway [55]. From these, the dehydrogenase pathway was identified by ^13^C tracer studies as major contributor to the overall lysine flux at high ammonium levels (Figure 1B, Additional file 1) which are readily present in production processes at industrial scale. The *ddh* gene, encoding diaminopimelate dehydrogenase, was hence chosen as integration site for genome-based implementation of the ectoine gene cluster. The intention was to lower the carbon flux via this pathway towards lysine to *a priori* diminish lysine formation as a major competitor for the building block ASA (Figure 1).

### Design of the genetic construct for host engineering

For our recombinant DNA experiments, we chose an *ectABCD* gene cluster from an isolate of *Pseudomonas stutzeri*[56], a well-known ectoine and hydroxyectoine producer [33,37]. To adjust the codon usage of the *ect* gene cluster employed by the natural host strain *P. stutzeri* A1501 to that preferred by *C. glutamicum*, we chemically synthesized and codon-optimized the *ectABCD* genes (Additional file 2). We uncoupled the expression of the naturally osmotically inducible operon [33] from osmotic-stress-derived signal transduction processes by positioning the synthetic *ectABCD* gene cluster under control of a strong and constitutive *C. glutamicum* promoter that is driving the expression of the structural gene (*tuf*) for the elongation factor Tu of *C. glutamicum*[57]. The resulting *tuf*_*p*_-*ectABCD* construct was additionally provided at its 5’- and 3’-ends with regions flanking the non-essential diaminopimelate dehydrogenase gene (*ddh*) for its targeted integration into the chromosome of *C. glutamicum* by a double-homologous recombination event (Figure 1A). Successful integration and inactivation was verified by PCR analysis and determination of the DDH activity, which was found absent in the novel strain. In this way, a recombinant *C. glutamicum* strain ECT-1 was constructed that carried a single-copy of the *tuf*_*p*_-*ectABCD* gene cluster stably integrated into a well-defined site in the chromosome and that should be able to produce ectoine/hydroxyectoine in the absence of osmotic stress.

### The recombinant *C. glutamicum* strain efficiently produces ectoine from glucose

**Figure 2:**
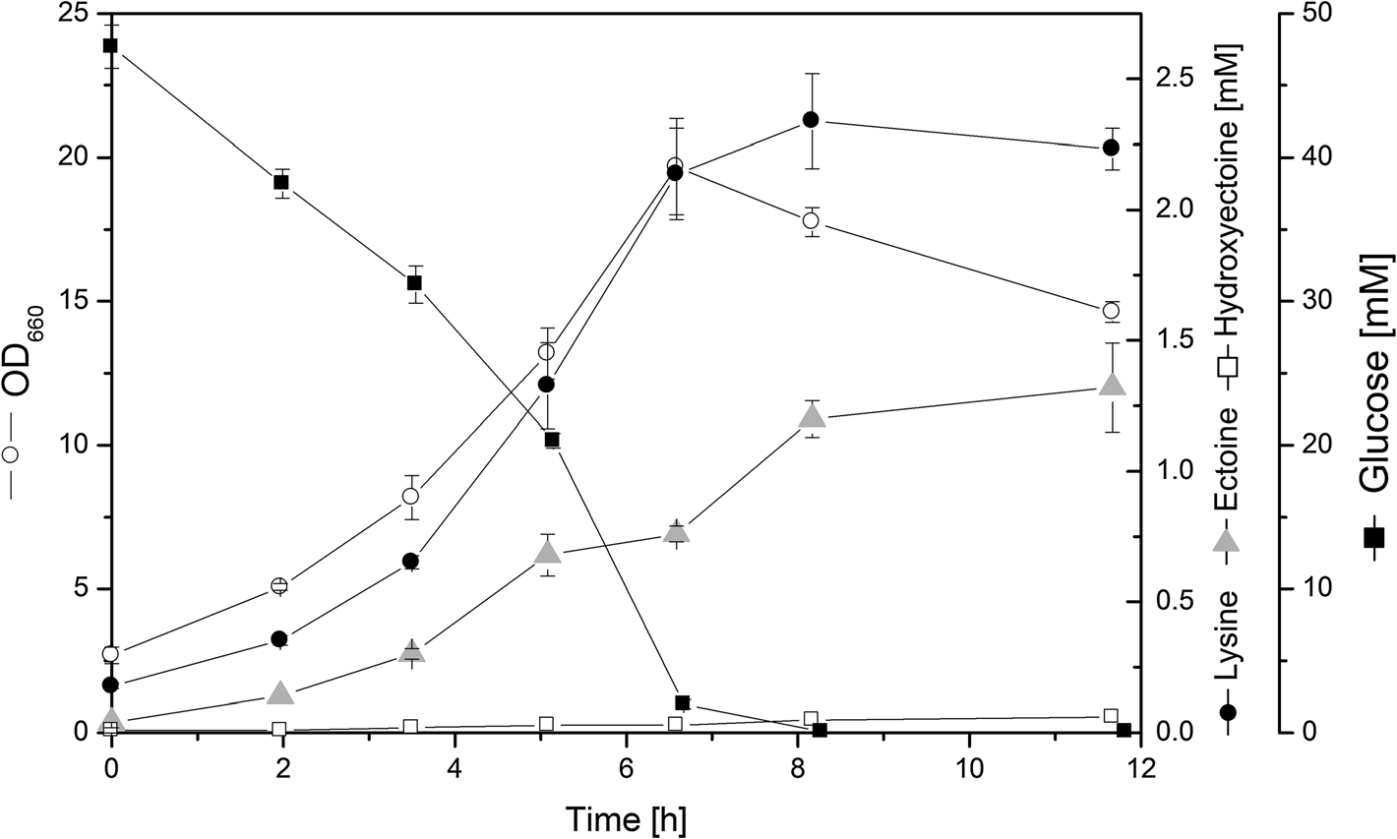
**Cultivation profile of the heterologous ectoine producer strain *C. glutamicum *ECT-1.** The *C. glutamicum* strain ECT-1 was cultivated in shake flasks at 30°C in a chemically defined medium. At the indicated time intervals, consumption of glucose and the extracellular accumulation of L-lysine, ectoine, and 5-hydroxyectoine were monitored. The data shown represent mean values and corresponding standard deviations from three biological replicates.

To evaluate the pattern and levels of ectoine/hydroxyectoine production in the newly constructed ECT-1 strain, we grew it in a chemically defined minimal medium with glucose as the carbon source. This experiment revealed that the *tuf*_*p*_-*ectABCD* gene cluster was functionally expressed, and we observed that the ECT-1 strain produced and secreted ectoine in shake-flask cultures already from the early growth phase (Figure 2). Ectoine accumulated in the culture supernatant up to a final titer of 1.3 mM after the cells had completely depleted their carbon source. About 2% of the consumed glucose was converted into ectoine as reflected by the molar yield of 19.4 mmol mol^-1^ (Table 1). Hydroxyectoine was also detected in the culture supernatant, albeit at a rather low level (< 0.1 mM). In addition to both ectoines, strain ECT-1 also secreted substantial amounts of L-lysine into the medium, 2.3 mM in total. L-lysine production thus exceeded that of the desired products ectoine and hydroxyectoine. We also quantified the intracellular concentrations of ectoine and hydroxyectoine in strain ECT-1. This revealed that ectoine accumulated up to 130 μmol (g_cdm_)^-1^ in the cytosol. Hydroxyectoine was detected in significantly lower amounts (~ 5 μmol (g_cdm_)^-1^). Hence, the relative amounts of these two compounds found inside the cells corresponded roughly to the different production titers measured for ectoine and hydroxyectoine in the supernatant (Figure 2).

**Table 1:** **Growth and production performance of the *C. glutamicum *** **strains LYS-1 [53], ECT-1 and ECT-2 during batch cultivation on a mineral salt medium with glucose as carbon source at 30°C (LYS-1, ECT-1, ECT-2) and 35°C (ECT-2)**

	***C. glutamicum *** **LYS-1**	***C. glutamicum *** **ECT-1**	***C. glutamicum *** **ECT-2**	***C. glutamicum *** **ECT-2**
**Temperature**	**30°C**	**30°C**	**30°C**	**35°C**
**Rates**				
μ [h^-1^]	0.38 ± 0.01	0.34 ± 0.00	0.36 ± 0.00	0.34 ± 0.02
q_s_ [mmol g^-1^ h^-1^]	4.86 ± 0.10	3.82 ± 0.08	3.51 ± 0.05	3.74 ± 0.10
q_Ect_ [mmol g^-1^ h^-1^]	-	0.07 ± 0.01	0.09 ± 0.01	0.12 ± 0.01
q_Lys_ [mmol g^-1^ h^-1^]	0.39 ± 0.02	0.20 ± 0.01	0.00 ± 0.00	0.00 ± 0.00
**Yields**				
Y_X/S_ [g mol^-1^]	82.1 ± 1.3	87.2 ± 2.8	101.5 ± 0.6	90.9 ± 6.3
Y_Ect/S_ [mmol mol^-1^]	-	19.4 ± 1.5	24.6 ± 0.6	32.0 ± 0.8
Y_Lys/S_ [mmol mol^-1^]	81.2 ± 3.2	53.8 ± 2.5	0.0 ± 0.0	0.0 ± 0.0
Y_Tre/S_ [mmol mol^-1^]	9.4 ± 0.4	5.6 ± 0.1	7.2 ± 0.9	6.7 ± 0.2
Y_AKG/S_ [mmol mol^-1^]	0.0 ± 0.0	2.6 ± 0.4	1.0 ± 0.1	1.9 ± 0.1
Y_EctOH/S_ [mmol mol^-1^]	-	0.6 ± 0.0	0.6 ± 0.0	0.9 ± 0.1

The data represent mean values and standard deviations from three biological replicates and denote the specific rates for growth (μ), substrate uptake (q_S_), and product formation (q_P_). Additionally, the yield for biomass (Y_X/S_), ectoine (Y_Ect/S_), lysine (Y_Lys/S_), trehalose (Y_Tre/S_), α-ketoglutarate (Y_AKG/S_), and hydroxyectoine (Y_EctOH/S_) are given.

### Increased temperature positively affects ectoine and hydroxyectoine production

**Figure 3:**
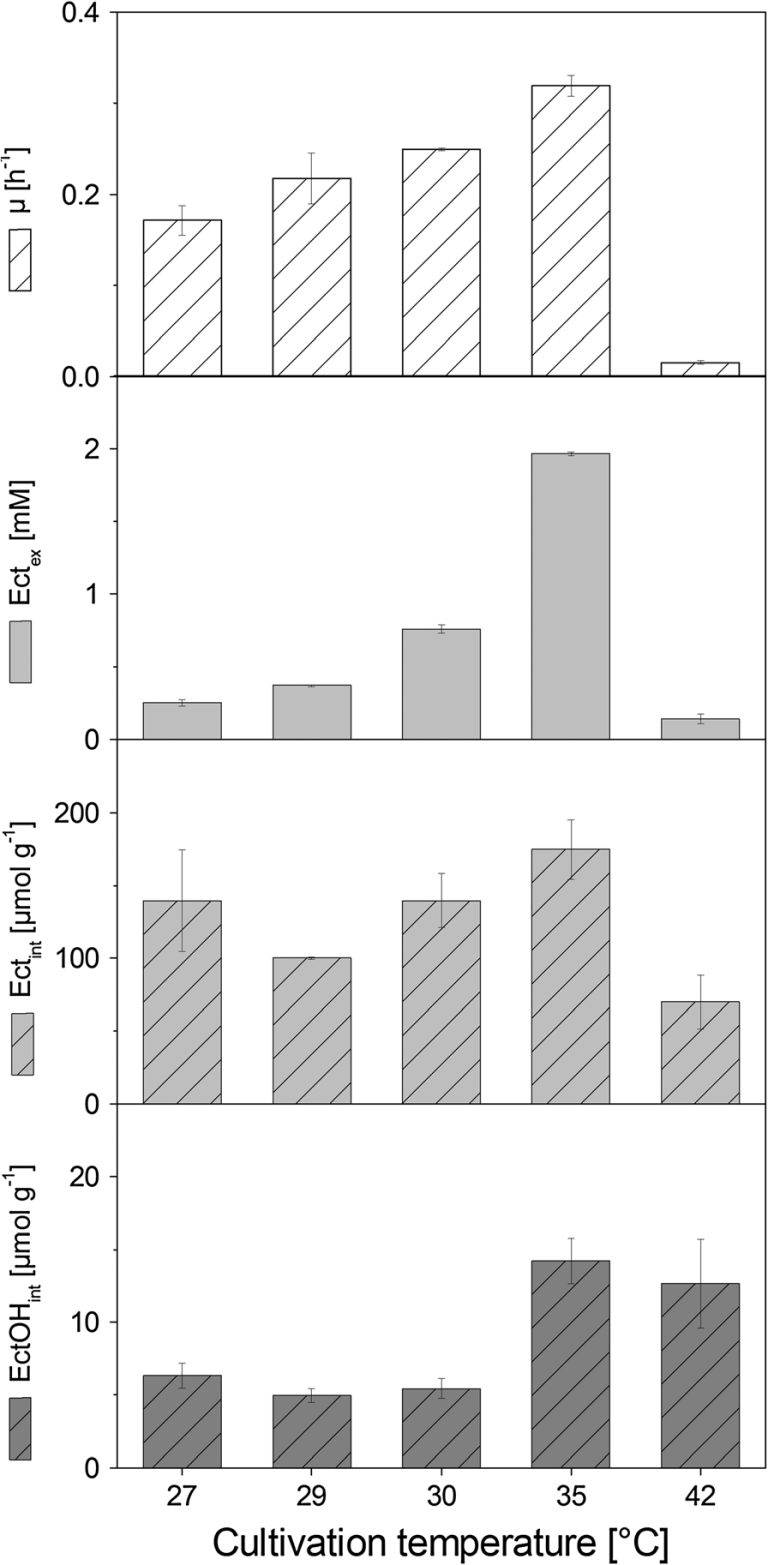
**Influence of cultivation temperature on the growth and ectoine production performance of *C. glutamicum *ECT-1.** Strain ECT-1 was grown in chemically defined medium with glucose on a miniaturized scale at the indicated growth temperatures. The specific growth rate μ, ectoine secretion (Ect_ex_), and intracellular accumulation of ectoine (Ect_int_) and hydroxyectoine (EctOH_int_) were determined. Ectoines were quantified after 10h (27°C, 30°C, 35°C) and 20h (42°C) of cultivation. The data shown represent mean values and corresponding standard deviations from three biological replicates.

The optimal cultivation temperature for the recombinant production of ectoine and hydroxyectoine by strain ECT-1 was assessed by miniaturized cultivations in a temperature range between 27°C and 42°C. Interestingly, this revealed that ectoine production was improved by higher temperature (Figure 3). As compared to the reference cultivation conditions for strain ECT-1 at 30°C, secretion was more than doubled when the temperature was increased to 35°C. The enhanced production performance was also reflected by a slight increase of the intracellular ectoine level. The higher cultivation temperature also positively influenced the intracellular amounts of hydroxyectoine, and the growth performance of strain ECT-1 as reflected by a 28% increase of the specific growth rate (Figure 3). The higher ectoine concentration in the supernatant was taken as positive indication for a better production performance at 35°C.

### Elimination of lysine secretion generates a second-generation ectoine producer

Ectoine production of the first-generation producer ECT-1 was limited by substantial carbon loss related to L-lysine synthesis and subsequent secretion of this amino acid into the growth medium. To avoid excretion of L-lysine, the gene (*lysE*) for the L-lysine exporter LysE [58] was inactivated in the genetic background of strain ECT-1 to yield the second-generation ectoine producer *C. glutamicum* ECT-2. Validation was carried out by PCR as previously described [58]. First, the novel ECT-2 strain was cultivated in shake flasks in glucose minimal medium at 30°C to allow a direct comparison of the performance with the parent ECT-1 strain. We found that the deletion of the *lysE* gene had a beneficial influence on ectoine production. As compared to the parent strain ECT-1, the molar ectoine yield was increased by 27% (Table 1). At the same time, L-lysine secretion was completely eliminated (Table 1). However, the additional carbon available for strain ECT-2 was not completely channeled towards ectoine production; it was instead recruited for biomass formation (Table 1). To take benefit from the improved ectoine production at elevated growth temperature observed for strain ECT-1 (Figure 3), we also investigated in greater detail the performance of strain ECT-2 at 35°C. Similarly to the ECT-1 strain, the higher growth temperature positively influenced the production performance of the strain ECT-2. The yield was increased from 25 mmol mol^-1^ at 30°C to 32 mmol mol^-1^ at 35°C. When compared to the basic proof-of-concept approach, the allover ectoine yield was improved in strain ECT-2 by 65%. In addition, strain ECT-2 did not suffer from high fluxes to the by-product L-lysine. We noted that the growth performance of strain ECT-2 was hardly affected by the elevated cultivation temperature (Table 1); however, the yield in biomass was reduced, likely as a stress response of *C. glutamicum* to the increase in growth temperature. Taken together, the substantially improved ectoine yield and the unaffected glucose uptake rate of strain ECT-2 resulted in an about 70% increased specific productivity of the second-generation ectoine producer *C. glutamicum* ECT-2 (Table 1).

### Ectoine synthesis affects the intracellular pools of amino acids of the aspartate family

ASA is an important metabolite and critical branch point with regard to biosynthesis of the aspartate family of amino acids [21]. Since ectoine biosynthesis is dependent on a good supply of ASA as well (Figure 1A) [32,33], we examined the intracellular pools of amino acids belonging to the aspartate family. Most desirable, integration of the synthetic ectoine cluster into the DDH lysine branch resulted in substantial decrease of the intracellular accumulation of lysine as major competitor in the ECT-1 strain (Table 2). This was taken as positive indication of a successfully lowered carbon flux towards lysine biosynthesis, thus increasing the ASA availability for the novel product ectoine. Ectoine indeed accumulated in substantially higher levels in the cytosol than lysine did (Table 2). Upon elimination of the lysine exporter, however, the metabolite pattern completely changed. The novel ECT-2 strain accumulated up to 77μmol (g_cdm_)^-1^ of lysine even exceeding that of the basic lysine producer LYS-1 by more than three fold. Simultaneously, the ectoine level dropped substantially (Table 2). Obviously, elimination of lysine secretion did not circumvent high carbon fluxes towards this amino acid but resulted in increased intracellular accumulation. In addition to the most obvious changes regarding the intracellular lysine and ectoine level, also the formation of aspartate and threonine was slightly affected. As compared to the parent lysine producer LYS-1, their cytosolic levels were slightly reduced in the synthetic ectoine cell factories. The intracellular asparagine level, however, was strongly increased. Whereas only marginal amounts were found in LYS-1, both ECT-1 and ECT-2 exhibited comparably high intracellular asparagine levels, also competing with ectoine biosynthesis and secretion. The cultivation temperature only had a marginal effect on the intracellular amino acid accumulation. For ectoine, a slight trend towards higher accumulation was observed at 35°C as indicated by the miniaturized temperature screening.

**Table 2:** **Concentration of free intracellular amino acids of the aspartate-family and of intracellular ectoine of the lysine-producing *C. glutamicum *** **strains LYS-1 and its ectoine-producing derivatives *C. glutamicum *** **ECT-1 and *C. glutamicum *** **ECT-2**

**Strain**	**Aspartate [μmol g**_**cdm**_^**-** **1**^**]**	**Asparagine [μmol g**_**cdm**_^**-1**^**]**	**Threonine [μmol g**_**cdm**_^**-1**^**]**	**Lysine [μmol g**_**cdm**_^**-1**^**]**	**Ectoine [μmol g**_**cdm**_^**-1**^**]**
(**A**)					
*C. glutamicum* LYS-1	11.8 ± 3.3	0.7 ± 0.3	5.1 ± 0.9	23.4 ± 3.1	--
*C. glutamicum* ECT-1	8.1 ± 2.9	24.6 ± 6.0	3.9 ± 0.6	14.5 ± 4.1	126.8 ± 25.5
*C. glutamicum* ECT-2	7.0 ± 0.3	20.1 ± 4.9	3.0 ± 0.9	76.7 ± 11.3	34.1 ± 14.2
(**B**)					
*C. glutamicum* LYS-1	9.1 ± 0.9	1.4 ± 0.2	6.8 ± 0.5	24.4 ± 6.3	--
*C. glutamicum* ECT-1	7.6 ± 0.6	28.1 ± 4.0	6.5 ± 0.4	16.3 ± 2.0	158.5 ± 20.7
*C. glutamicum* ECT-2	7.9 ± 0.6	17.0 ± 2.4	5.2 ± 0.4	52.8 ± 12.6	36.1 ± 7.6

Cells were grown at 30°C (A) and 35°C (B), respectively in mineral salt medium. The data represent mean values with standard deviations from two biological replicates, each sampled at three different optical densities (OD 2, OD 4 and OD 8).

### Performance of the ectoine-producing *C. glutamicum* strain ECT-2 under fed-batch conditions

**Figure 4:**
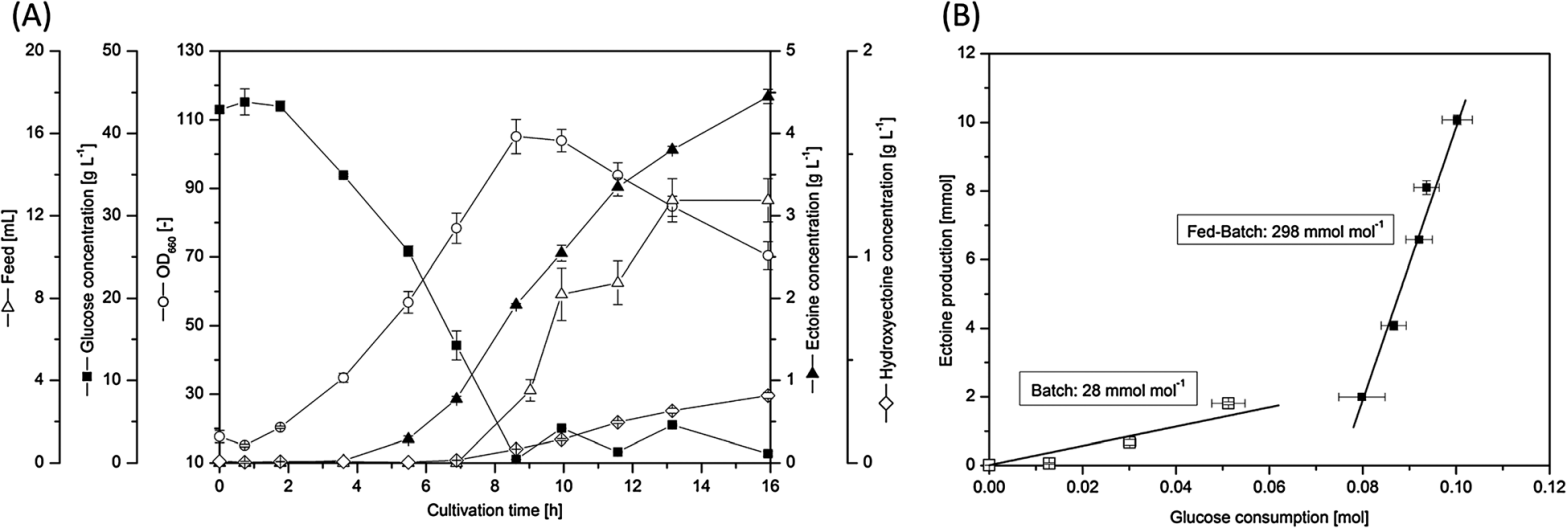
**Production performance of the advanced ectoine**-**producer strain *C. glutamicum *ECT**-**2 during fed**-**batch fermentation.** Cultivation profile of strain ECT-2 **(A)**, and ectoine yield achieved in the different cultivation phases **(B)** are shown. The oxygen saturation in the fermenter was kept constant at 30% by variation of the stirrer velocity and the aeration rate. Automated feeding was initiated by a pO_2_-based signal [53]. Glucose concentration was thereby kept below 5 g L^-1^. The data shown represent mean values from two independent fermentation experiments.

To assess the overall production performance of the ECT-2 strain under conditions more relevant for an industrial process, we benchmarked ectoine production by this strain in a fed-batch process. Ectoine was secreted from early on during cell growth and accumulated in the growth medium to a final concentration of 4.5 g L^-1^ after 16 h of fermentation (Figure 4A). We observed that the production efficiency differed significantly between the batch and the feeding phase of the process (Figure 4B). The initial 45 g L^-1^ glucose was already consumed after 8 h and mainly served for the production of biomass (Figure 4A). This was reflected by the rapid increase of the optical density to OD_660_ 100. As soon as the feeding was started, a shift in the production pattern towards the formation of ectoine was observed. Overall, the feeding phase contributed to more than 80% of the total ectoine production (Figure 4). The yield obtained during the batch phase (28 mmol mol^-1^) was very similar to the yield obtained during shake flask cultivation of strain ECT-2 (Table 1). In the feeding phase, however, it increased 10-fold up to ~300 mmol mol^-1^ (Figure 4B). This substantial increase provided an overall space time yield of 6.7 g L^-1^ ectoine per day.

## Discussion

The stabilizing and function-preserving effects of ectoines have spurred considerable interests in these compounds and have led to the development of a variety of practical applications [7,13,14]. As a consequence, ectoine and its derivative hydroxyectoine are considered as valuable natural microbial products and they have gained significant market value in recent years. Their biotechnological production has reached the scale of tons on an annual basis. The industrial production of ectoines is currently achieved by bacterial milking of *H. elongata *[8,20], a process that has recently been improved by the inclusion of mutants of this bacterium that cannot catabolize ectoines and those that accumulate these compounds in the high salinity medium as a result of a defect in the ectoine/hydroxyectoine-specific TeaABC uptake system [36]. We report here the construction and characterization of a synthetic microbial cell factory for the production of ectoine that relies on the robust metabolism of *C. glutamicum *[59,60]. This bacterium incorporates features desirable for large scale production processes which are responsible for its tremendous rise and success as industrial production host [46].

### Novel *C. glutamicum* designer-bug enables decoupling of ectoine production from high salinity in a marker-free host system

We build on the considerable knowledge base for the genetic manipulation and large-scale fermentation processes of the industrial workhorse *C. glutamicum *[46]. Most advantageous, the *C. glutamicum* genome sequence lacks ectoine catabolic genes [61] eliminating product loss due to the reuse of ectoine and hydroxyectoine as carbon or nitrogen sources - a drawback of natural producer *H. elongata *[36]. In developing the synthetic *C. glutamicum* ectoine cell factory several strategies were combined simultaneously to optimize its production. Adaptation of the codon usage appeared promising as this strategy has recently proven beneficial for the heterologous production of diaminopentane recruiting lysine decarboxylase from *E. coli *[62]. Expression control via the constitutively active *tuf* promoter of *C. glutamicum *[57,62] not only decoupled *ectABCD* expression from its natural osmo-stress responsive regulation [33] but also provided a simple and robust promoter system with no specialized needs for the control of its activity. Beyond this, the careful design of the cellular chassis guaranteed good supply of the building block ASA which is normally tightly controlled in many microorganisms including *C. glutamicum *[21,54]. In addition, and in an effort to diminish carbon flux towards L-lysine, the dehydrogenase branch of L-lysine biosynthesis [63,64] was inactivated (Figure 1A, B). In comparison to plasmid-based systems [33,37,43,44] our strategy benefits from a stable genome-based integration of the synthetic *tuf*_*p*_-*ectABCD* gene cluster (Figure 1B), an approach that has also been employed for the recombinant production of ectoines by *H. polymorpha *[45]. However, high-copy plasmids could provide increased expression levels of the biosynthetic genes which appears to be limiting the production efficiency at least of the ECT-2 strain. Towards industrial scale production, multiple genome-integrated gene copies and optimization of ribosomal binding sites appear particularly attractive. Constitutive gene expression in the synthetic *C. glutamicum* cell factory obviated the need for the use of high-salinity growth media, conditions that are typically required to trigger enhanced expression of these genes in natural producers [24,26,27] such as *H. elongata*[8]. Our synthetic cell factory thus avoids the considerable drawbacks of high-salinity growth media during fermentation processes that invoke corrosion problems on the reactor systems [44] and thereby drive up the costs for their appropriate design and maintenance. Related to the economic relevance, also previous studies have addressed the issue of a salt-decoupled ectoine production with the natural producers *P. stutzeri* and *Chromohalobacter salexigens*, and the heterologous hosts *H. polymorpha* and *E. coli *[37,43-45].

### Combined process and strain engineering increases ectoine production in *C. glutamicum*

As successful proof of concept, the first generation *C. glutamicum* ECT-1 strain revealed salinity-decoupled ectoine and hydroxyectoine production and secretion. Interestingly, the summed molar yields of L-lysine and ectoine added up to 73.2 mmol mol^-1^, a value that perfectly matches with the L-lysine yield of the parent strain LYS-1 [53]. Hence, the carbon demand for ectoine synthesis seems to be completely satisfied by the cells on the expense of L-lysine. Still, L-lysine production predominated in the basic ectoine producer. This issue was addressed in a second round of metabolic engineering through inactivation of the gene coding for the L-lysine exporter LysE. This strategy has recently been applied for optimized diaminopentane production by *C. glutamicum *[65]. Beyond the 27% gain in ectoine yield (Table 1), the inactivation of the lysine exporter avoided a contamination of the excreted ectoine, a feature that will clearly facilitate the downstream processing for the recovery of ectoine from the culture broth. Further improved ectoine production was then achieved by an increase in the growth temperature from 30°C to 35°C. In combination, genetic and process engineering proved to be highly beneficial as reflected in a 65% increased yield and a 70% increased specific productivity (Table 1). A rather unexpected finding of our study was the observation that the recombinant strain produced so little hydroxyectoine (Table 1). This is surprising because *P. stutzeri* A1501 on whose genetic blueprint we build our synthetic *ectABCD* gene cluster produces hydroxyectoine very efficiently and in much greater quantities than ectoine [33,37]. We can currently not comment about why there is so little hydroxyectoine formed by the *C. glutamicum* [*tuf*_*p*_-*ectABCD*] strains. Overall, both recombinant *C. glutamicum* ectoine producers already performed admirably in shake-flask experiments. They exhibited excellent specific production rates of 9.9 mg g^-1^ h^-1^ (ECT-1) and 17.1 mg g^-1^ h^-1^ (ECT-2), thus exceeding that of the native and the heterologous ectoine production hosts *H. elongata* (7.1 mg g^-1^ h^-1^) [20] and *E. coli* (2 mg g^-1^ h^-1^) [43]. However, a closer examination of the finally achieved yield of 32 mmol mol^-1^ revealed, that it was so far not possible to harness the complete carbon used by *C. glutamicum* LYS-1 for L-lysine synthesis for the recombinant production of ectoine.

### High-cell density fermentation reveals excellent production performance

To take benefit from high cell densities [53,66] that cannot readily be achieved in shake-flask experiments we benchmarked the performance of the second generation ectoine producer *C. glutamicum* ECT-2 under carefully controlled fed-batch conditions. We found that the ectoine production efficiency differed significantly during the batch and the feeding phase of the fermentation process. That kind of desired shift in the product spectrum is often intentionally induced at industrial scale production by appropriate process operations. In general, this leads to an optimized channeling of the substrate to the desired product within the feeding-phase [53,67-69]. Similarly, we observed an admirable 10-fold increase of the ectoine yield in the feeding phase of the ECT-2 strain fermentation (Figure 4B). Though there was detectable growth-associated ectoine production during the batch phase, the predominant fraction of ectoine seemed to be produced by cells during the feeding phase. The finally achieved titer of 4.5 g L^-1^ already approached to that of currently described industrial production systems [38,39,41]. The *C. glutamicum* cell factory, however, takes high benefit from the fast growth and vitality. The excellent performance of the *C. glutamicum* strain ECT-2 under fed-batch conditions allowed the synthetic cell factory to achieve an overall space-time yield of 6.7 g L^-1^ ectoine per day which is among the highest productivities reported so far in the literature [38,39,41]. Better performance was, so far, only achieved with *H. boliviensis*[38], and *Chromohalobacter salexigens *[41]. These do, however, rely on high salinity and involve complex process operation strategies [41] thus driving up the production costs.

### Metabolic changes point at potential bottlenecks in ectoine production

When introducing the synthetic ectoine gene cluster in lysine producing *C. glutamicum* LYS-1, carbon flux within the cell was partly rerouted from the common intermediate ASA to ectoine as indicated by the drop in extracellular and intracellular lysine ([53], Table 2), an effect equally observed at 30°C and 35°C. However, elimination of lysine secretion resulted in a strong increase of the intracellular pool of this amino acid. The additional carbon, now potentially elevating the ASA pool, was not efficiently recruited for driving ectoine biosynthesis. Obviously, the native lysine route is still superior to the non-native route towards the novel product ectoine despite the elimination of the dehydrogenase branch. In subsequent rounds of strain engineering, this might be targeted by additional down-regulation of other lysine biosynthetic genes including dihydrodipicolinate synthase (*dapB*) and diaminopimelate decarboxylase (*lysA*) previously identified as key reactions for lysine production [53]. In addition, manipulating the strength of the ribosome-binding sites of the various *ect* genes might improve their expression. An additional issue involves the transport processes related to the ectoine metabolism. Most favorable for our production process *C. glutamicum* excretes ectoine into the growth medium, a feature that was also observed for other recombinant ectoine production systems [43,45]. So far the intracellular metabolite levels (Table 2) do not suggest limitations in the transport processes but experience has shown that engineering the excretion systems for amino acids and related compounds is beneficial for the performance of *C. glutamicum *[58,65,70-73]. In addition, it appears even important to address the uptake system of *C. glutamicum* comprising the three transporter EctP, LcoP, and ProP [74]. In native producers, these transport systems not only serve for the scavenging of these stress protectants from natural resources [75], but they also function as salvaging systems to retrieve compatible solutes that leak or are actively released from the producer cells [36,76,77]. As previously shown for the production of aromatic amino acids [78,79], the production efficiency of *C. glutamicum* might significantly suffer from product re-uptake into the cell. In addition, there is strong indication that the compatible solute transporter TeaABC in *H. elongata* is integrated in the regulation of ectoine biosynthesis. *De novo* biosynthesis of the cell is immediately decreased when externally supplied ectoine is taken up via TeaABC [36]. As the regulatory mechanism is so far unknown, similar regulation patterns involving the *C. glutamicum* ectoine transporters might also limit the biosynthetic efficiency in the recombinant production host.

## Conclusion

In contrast to the currently used *H. elongata* strain for the commercial production of ectoines [7,8], or other microbial species that have been suggested as alternative natural producers [33,37-40], *C. glutamicum* has a long history for the industrial level production of valuable natural products by large-scale fermentation procedures [73,80]. Knowledge gained during the development of *C. glutamicum* into an excellent performing microbial cell factory [53,68,81] can now be brought to bear in a scale-up process for ectoine production by the recombinant *C. glutamicum* [*tuf*_*p*_-*ectABCD*] ECT-2 strain. In addition, the ability to readily manipulate the genetic blueprint of *C. glutamicum* on a genome-wide scale [50,53,82] will allow the application of rational and systems-wide metabolic engineering approaches to further improve the performance of the second generation ectoine producer ECT-2. The success of these approaches has recently unequivocally been demonstrated in the development of highly efficient L- lysine [53] and diaminopentane [62,68,83]*C. glutamicum* cell factories.

## Materials and methods

### Microorganisms and plasmids

In the present work, the ATCC 13032-derived L-lysine-overproducing *C. glutamicum* strain LYS-1 [53] was used as host for ectoine and hydroxyectoine production. The strains and plasmids employed in this study are listed in Table 3. Amplification of transformation vectors during genetic engineering work was carried out in *Escherichia coli* strains DH5α and NM522 (Invitrogen, Carlsbad, CA, USA) [53].

**Table 3:** **Description of the *C glutamicum *** **strains and plasmids used in the present work for heterologous production of ectoine and hydroxyectoine**

**Strain**	**Description**	**Reference**
*C. glutamicum* LYS-1	*C. glutamicum* ATCC 13032 + amino acid exchange T311I in the *lysC* gene (NCgl0247), encoding aspartokinase	[53]
*C. glutamicum* ECT-1	LYS-1 + genome-based integration of the codon-optimized biosynthetic ectoine cluster of *P. stutzeri* into the *ddh* gene (NCgl2528), encoding diaminopimelate dehydrogenase [ddh: *tuf*_*p*_-*ectABCD*]	This work
*C. glutamicum* ECT-2	ECT-1 + deletion of *lysE* gene (NCgl1214), encoding the lysine exporter	This work
**Plasmids**	**Description**	**Reference**
pTC	Expression vector for DNA-methyltransferase of *C. glutamicum*, ORI for *E. coli* and tetracycline resistance as selection marker. Used in *E. coli* NM522 to add the *C. glutamicum*-specific DNA-methylation pattern to the integrative transformation vector.	[53]
pClik int *sacB*	Integrative transformation vector for *C. glutamicum* with MCS, ORI for *E. coli*, and Kan^R^ and *sacB* as selection markers.	[57]
pClik int *sacB ectABCD*	Integrative transformation vector for the integration of the codon-optimized biosynthetic ectoine/hydroxyectoine gene cluster derived from *P. stutzeri* A1501 into the *ddh* locus of *C. glutamicum*	This work
pClik int *sacB* Δ*lysE*	Integrative transformation vector for deletion of the lysine exporter *lysE*	[65]

### Strain construction

The heterologous production of ectoine in *C. glutamicum* was based on the *ectABCD* biosynthetic gene cluster from *Pseudomonas stutzeri* A1501 [33]. We attached to the 5’ end of the *ectABCD* gene cluster a 200-bp DNA segment that carries the strong and constitutively active promoter driving the expression of the structural *tuf* gene (NCgl0480) of the elongation factor Tu of *C. glutamicum *[57]. Additionally, the construct was flanked by ~ 560 bp-sized homologous recombination sites for genome-based integration of the construct into the structural *ddh* gene (NCgl2528), encoding diaminopimelate dehydrogenase. Artificial *Spe*I digestion sites were added at the 5’ and 3’ end. The 4577 bp-sized construct (Figure 1A) was synthesized by Geneart (Life Technologies, Regensburg, Germany), whereby the codon usage was adjusted to that preferred by *C. glutamicum* using the proprietary GeneOptimizer® software (Geneart, Life Technologies, Regensburg, Germany). The DNA sequence of the codon-optimized *ectABCD* variant gene cluster along with the original sequence of *P. stutzeri* A1501 is given in the supplement. Insertion of the synthetic construct containing the *ectABCD* gene cluster into the integrative transformation vector pClik_int_*sacB *[57] was carried out at the unique *Spe*I site. Vector amplification in the *E. coli* strains DH5α and NM522, purification of plasmid DNA, and its transformation into *E. coli* and *C. glutamicum* strains were performed as described previously [84]. The codon-optimized *ectABCD* gene cluster was inserted by DNA transformation into the ATCC 13032-derived *C. glutamicum* strain LYS-1 carrying a feedback deregulated aspartokinase [85]; this yielded the recombinant *C. glutamicum* strain ECT-1. The correct integration into the chromosome of strain ECT-1 at the non-essential diaminopimelate dehydrogenase gene (*ddh*) was verified by PCR using the *ddh*-specific primers ECT_fw (5’-AGAGTACCTGGGACGCAGCGTCG-3’) and ECT_rev (5’- CGTCGCGTGCGATCAGATCGGT-3‘) and a KAPAHiFi PCR-Kit (Peqlab, Erlangen, Germany). The PCRs were performed under conditions recommended by the manufacturers of the PCR-Kit. The PCR yielded the expected ~ 3.5-kb product comprising the synthetic *P. stutzeri ectABCD* genes fused to the *C. glutamicum tuf* promoter (Figure 1). Concomitant inactivation of the chromosomal *ddh* gene was verified in the *C. glutamicum* ECT-1 strain by assaying diaminopimelate dehydrogenase (DDH) enzyme activity. The parent *C. glutamicum* LYS-1 strain exhibited a DDH enzyme activity of 300 mU mg^-1^, whereas no DDH enzyme activity (< 0.1 mU mg^-1^) was detected in the recombinant *ddh*::*tuf*_*p*_-*ectABCD C. glutamicum* strain ECT-1. A deletion of the gene (*lysE*) encoding the L-lysine exporter of *C. glutamicum* was introduced into the genome of strain ECT-1 as described previously [65]; the resulting strain was named *C. glutamicum* ECT-2.

### Cultivation of *C. glutamicum* strains in shake flasks

The preparation of the inoculum involved two subsequent pre-cultivation steps using either complex or glucose-based minimal medium [62,86]. The main cultivation was then carried out in a chemically defined mineral salt medium with glucose as the carbon source [84]. If not specified otherwise, *C. glutamicum* was grown in baffled shake flasks with a 10% filling volume at 30°C and 230 rpm on an orbital shaker (Multitron, Infors AG, Bottmingen, Switzerland; 5 cm diameter). For ^13^C labeling experiments, the naturally labeled glucose was replaced by an equimolar amount of 99% [3-^13^C]-glucose (Cambridge Isotope Laboratories, Inc., Andover, MA, USA). The tracer studies were carried out at an ammonium sulfate concentration of 5 g L^-1^ and 50 g L^-1^, respectively, to study the split ratio of the branched lysine biosynthesis.

### Miniaturized cultivation of *C. glutamicum* strains

Small-scale cultivations of *C. glutamicum* strains were carried out in 1 mL mineral salt medium in 48-well flower plates (m2p-labs, Baesweiler, Germany) at 700 rpm using the Biolector system (DASGIP, Jülich, Germany). The temperature was varied between 27°C and 42°C. To avoid evaporation of the medium, the plates were sealed with a gas-permeable membrane (Aera Seal, Sigma Aldrich, Steinheim, Germany). Inoculum preparation involved shake flask cultivation as described above, and these cells were then subsequently used to inoculate the multi-well plates to an initial optical density of 0.3. Cell growth was monitored online via measurement of the optical density at 620 nm. After 10 h (27°C, 30°C, 35°C) and 20 h (42°C), extracellular and intracellular accumulation of ectoines was determined. Process monitoring, data collection, and data processing were carried out with the software suit BioLection (m2p-labs, Baesweiler, Germany).

### Fed-batch cultivation for ectoine production in *C. glutamicum*

Production performance of *C. glutamicum* ECT-2 was investigated by fed-batch cultivation in (1000 mL) bioreactors with 300 mL starting volume (SR0700ODLS, CWD4 Bioblock, DASGIP AG, Jülich, Germany). The reactors were equipped with a pH electrode (Mettler Toledo, Giessen, Germany) and a pO_2_ electrode (Hamilton, Höchst, Germany). Data acquisition and online control of the process were carried out with the DASGIP control software. The pH was kept constant at 6.9 by automated addition of 25% NH_4_OH (MP8 pump system, DASGIP AG, Jülich, Germany). Dissolved oxygen was maintained at saturation above 30% by variation of the stirrer speed and the aeration rate; these values were initially set to 0.5 vvm and 400 rpm, respectively. The temperature was kept constant at 35°C (CWD4 Bioblock, DASGIP AG, Jülich, Germany). The feeding was automatically controlled via the dissolved oxygen level [53]. Inoculum preparation involved pre-cultivation for 10 hours in the fermentation batch medium (MMYE-1 medium [87]), and cells were harvested by centrifugation (5350 × *g*, 10 min, Heraeus Multifuge 4KR, Thermo Fisher Scientific, Schwerte, Germany). The cultivation of the cells was carried out in MMYE-1 medium [87] without adding homoserine. For preparation of the feeding solution, the glucose and ammonium sulfate concentrations were increased to 350 g L^1^ and 50 g L^-1^, respectively.

### Sample preparation for metabolite quantification

For the quantification of glucose and of secreted products, the biomass was separated from the culture broth by centrifugation (13000 × *g*, 5 min, 4°C and 10000 × *g*, 15 min, 4°C, centrifuge 5415R Eppendorf, Hamburg, Germany). For the quantification of intracellular concentrations of ectoine and hydroxyectoine, cells from 1 mL culture were harvested by centrifugation (13000 × *g*, 5 min, 4°C). The exact sample volume was determined gravimetrically on an analytical balance (CP255D, Sartorius, Göttingen, Germany). In parallel, the optical density was measured to quantify the harvested biomass amount. The supernatant was decanted, and the cells were subsequently dried for 12 h in a speedvac apparatus (Concentrator 5301, Eppendorf, Hamburg, Germany). Cells were re-suspended in 500 μL Bligh & Dyer solution (MeOH:CHCl_3_:H_2_0, 10:5:2) and disrupted mechanically (FastPrep®-24, 3 min, 4 m s^-1^, MP Biomedicals, Santa Ana, USA) using glass beads with a diameter of 0.04 mm. The disruption step was repeated, after adding 130 μL H_2_O and 130 μL chloroform to the slurry. Phases were then separated by centrifugation (10000 × *g*, 15 min centrifuge 5415R, Eppendorf, Hamburg, Germany). The aqueous phase was transferred into a clean reaction tube and subsequently evaporated to dryness in a speedvac apparatus. The remaining solids were dissolved in 80–100 μL H_2_O and centrifuged (15 min, 10000 × *g*, 4°C); metabolites were then assessed by HPLC analysis. Sampling for quantification of the intracellular amino acid concentration was performed by fast filtration [88].

### Substrate and product analysis

Glucose was quantified enzymatically in 1:10 diluted supernatant samples using the biochemical analyzer YSI 2700 Select (Kreienbaum, Langenfeld, Germany) [84]. Amino acids were quantified by HPLC (Agilent 1200 Series, SIM, Oberhausen, Germany) involving pre-column derivatization and fluorescence detection as described previously [89]. Quantification of ectoine and hydroxyectoine was performed by HPLC analysis (LaChrome, Merck-Hitachi, Darmstadt, Germany) using a ProntoSil C18 AQ + column (125 × 4 mm, Knauer, Berlin, Germany) with a Nucleosil C18 AQ + pre-column (120 × 5 mm, Knauer, Berlin, Germany). As mobile phase, a phosphate buffer was used (0.8 mM K_2_HPO_4_; 6.0 mM Na_2_HPO_4_, pH 7.6) at a flow rate of 1 mL min^-1^ and at 40°C. The injection volume was 2 μL. Detection was carried out with a diode array detector (L7450, LaChrome, Merck-Hitachi, Darmstadt, Germany) at 220 nm. Quantification of cell concentration was carried out by measuring the optical density (OD_660_). The cell dry mass was determined as described previously [89].

### GC-MS labeling analysis

Quantification of the ^13^C labeling of proteinogenic amino acids and of diaminopimelate from hydrolyzed biomass was carried out by GC-MS (GC 7890A, inert MSD 5979C, Agilent Technologies, Waldbronn, Germany) as described previously [90]. Derivatization and GC-MS measurement was then carried out as described for protein-bound amino acids [90]. Selected ion monitoring was performed for the [M-57] fragments for alanine (ALA, m/z 260), aspartate (ASP, m/z 418), lysine (LYS, m/z 431), and diaminopimelate (DAP, m/z 589), for the [M-85] fragment of alanine (m/z 232), and for the [M-159] fragment of aspartate (m/z 316), and lysine (m/z 329) (see also Additional file 1).

### Quantification of the flux-split ratio of the branched L-lysine pathway

The labeling data from GC-MS analysis were corrected for the natural isotopes [91] and used to calculate the molar ^13^C enrichment (ME) of the analyzed sample [92]. Related to the fragmentation pattern obtained by electron impact ionization of TBDMS-derivatized amino acid [92], the ^13^C enrichment of the C_1_-carbon in pyruvate (PYR), oxaloacetate (OAA), and LYS could be calculated from the [M-57] fragment, and the [M-85] fragment of alanine, and [M-159] fragment of ASP and LYS, respectively (Equations 1 – 3). Thirteen-C enrichment of DAP C_7_ was calculated from the [M-57] fragment of DAP and LYS, respectively (Equation 4). Further details are given in Additional file 1.

(1) $$ME_{\mathrm{PYR}\_C1}=ME_{\text{ALA}260}-ME_{\text{ALA}232}$$

(2) $$ME_{\text{OAA}\_C1}=ME_{\text{ASP}418}-ME_{\text{ASP}316}$$

(3) $$ME_{\text{LYS}\_C1}=ME_{\text{LYS}431}-ME_{\text{LYS}329}$$

(4) $$ME_{\text{DAP}\_C7}=ME_{\text{DAP}589}-ME_{\text{LYS}431}$$

Considering the carbon transition of the two parallel biosynthetic pathway branches in *C. glutamicum* ([63], Additional file 1), the contribution of the dehydrogenase branch to the allover L-lysine flux was calculated as follows (Equations 5, 6):

(5) $$f_{\text{DH}}=\frac{ME_{\text{LYS}\_C1}-ME_{\text{DAP}\_C7}}{ME_{\mathrm{DAP}\_C7}+ME_{\mathrm{LYS}\_C1}-2\times ME_{\mathrm{PYR}\_C1}}$$

(6) $$f_{\text{DH}}=\frac{ME_{\text{LYS}\_C1}-ME_{\text{DAP}\_C7}}{2\times ME_{\text{OAA}\_C1}-ME_{\text{LYS}\_C1}-ME_{\text{DAP}\_C7}}$$

### Determination of diaminopimelate dehydrogenase activity

Preparation of crude cell extracts was carried out by mechanical cell disruption and removal of cell debris by centrifugation [86]. The protein content was then determined by the method of Bradford [93]. Diaminopimelate dehydrogenase (DDH) activity was measured in the direction of the DDH-catalyzed oxidation of *meso*-diaminopimelate and concomitant NADPH formation; this enzyme assay required an alkaline pH of 10.5 [64]. The final reaction mixture contained 200 mM glycine/NaOH (pH 10.5), 10 mM MgCl_2_, 2 mM NADP, 8 mM *meso*-diaminopimelate, and 50 μl of crude cell extract. The change in absorbance of the enzyme assay mixture was monitored online at 340 nm (Specord 40; Analytik Jena, Jena, Germany). Negative controls were performed as previously described [57].

## Abbreviations

AcCoA: Acetyl-CoA; ALA: Alanine; AKG: α-ketoglutarate; ASA: L-aspartate-β-semialdehyde; Asd: L-aspartate-β-semialdehyde dehydrogenase; Ask: aspartokinase; ASP: Aspartate; CoA: Coenzyme A; DAP: Diaminopimelate; DDH: Diaminopimelate dehydrogenase; Ect: Ectoine; EctA: 2,4-diaminobutyrate acetyltransferase; EctB: L-2,4-diaminobutyrate transaminase; EctC: Ectoine synthase; EctD: Ectoine hydroxylase; EctOH: Hydroxyectoine; fDH: Relative flux through DDH; Glu: Glutamate; KanR: kanamycin resistance; LYS: Lysine; LysE: Lysine exporter; MCS: Multiple cloning site; ME: Molar enrichment; OAA: Oxaloacetate; ORI: Origin of replication; PCR: Polymerase chain reaction; PYR: Pyruvate; sacB: Encoding gene of levansucrase from *Bacillus subtilis*; Suc: Succinate; TBDMS: Tertbutyl-dimethyl-silyl; Tre: Trehalose; Tuf: Elongation factor tu.

## Competing interests

The authors declare no competing interests.

## Authors’ contribution

NS and NSB performed genetic engineering of the ECT-1 strain. NSB conducted cultivation experiments with ECT-1 in shake flasks and at miniaturized scale. BJH constructed the ECT-2 strain. BJH and RS performed shake flask cultivations with ECT-2. RS, BJH and MK carried out fed-batch fermentation and metabolite studies. JB conducted labeling experiments. JB and RS designed experiments. JB, EB and CW conceived and structured the work, assessed the data and wrote the manuscript. All authors read and approved the manuscript.

## Supplementary Material

Additional file 1 Flux split quantification of lysine pathway. Click here for file

Additional file 2 Gene_cluster_alignment. Click here for file
