# Supplementary material for: Systems metabolic engineering of Corynebacterium glutamicum for production of the chemical chaperone ectoine
Source: Microb Cell Fact. 2013 Nov 15;12:110. doi: 10.1186/1475-2859-12-110 (PMC4225761; doi:10.1186/1475-2859-12-110)
Supplement: Additional file 1 — Flux split quantification of lysine pathway. [file 1475-2859-12-110-S1.pdf]

Supporting Information for

Systems metabolic engineering for production of the chemical chaperone ectoine in *Corynebacterium glutamicum*

by

Judith Becker[1], Rudolf Schäfer[1], Michael Kohlstedt[1], Björn J. Harder[1], Nicole S. Borchert[1], Nadine Stöveken[2,3], Erhard Bremer[2,3], Christoph Wittmann[1#]

[1] Institute of Biochemical Engineering, Technische Universität Braunschweig, Germany

[2] Department of Biology, Laboratory for Microbiology, Philipps-University Marburg, Germany

[3] LOEWE-Center for Synthetic Microbiology, Philipps-University Marburg, Germany

Quantification of the flux-split ratio of the branched L-lysine pathway

In this work, we applied a GC-MS based approach for quantification of the flux split ratio of the branched lysine pathway. This involved ^{13}C tracer studies using 99% [3-^{13}C] glucose and subsequent GC-MS analysis of a defined set of metabolites including alanine (ALA), aspartate (ASP), diaminopimelate (DAP) and lysine (LYS). The labeling data of ALA and ASP were used to deduce the labeling information of pyruvate (PYR) and oxaloacetate (OAA), which were required for calculating the flux split ratio. This chosen experimental set-up evokes a characteristic isotopomer pattern in LYS and DAP which depends on the flux split ratio of the lysine pathway as illustrated in Figure S1. The molar enrichment (ME) [1] of DAP_C7 and LYS_C1 thereby depends on the relative contribution of the dehydrogenase branch (f_{DH}) and the succinylase branch (f_{SUC}), and the molar enrichment (ME) of PYR_C1 and OAA_C1, respectively, (equations S1 and S2). This highly specific carbon transition has previously been used to determine the flux split ratio via NMR which directly provides information about the positional ^{13}C enrichment of a single carbon atom within the molecule [2].

$$ME_{DAP\_C7} = f_{DH} \times ME_{PYR\_C1} + \tfrac{1}{2} \times f_{SUC} \times ME_{PYR\_C1} + \tfrac{1}{2} \times f_{SUC} \times ME_{OAA\_C1} \qquad \textbf{eq. S1}$$

$$ME_{LYS\_C1} = f_{DH} \times ME_{OAA\_C1} + \tfrac{1}{2} \times f_{SUC} \times ME_{PYR\_C1} + \tfrac{1}{2} \times f_{SUC} \times ME_{OAA\_C1} \qquad \textbf{eq. S2}$$

Accordingly, the relative contribution of the DH branch to the overall lysine flux can be calculated as follows:

$$f_{DH} = \frac{ME_{LYS\_C1} - ME_{DAP\_C7}}{ME_{DAP\_C7} + ME_{LYS\_C1} - 2 \times ME_{PYR\_C1}} \qquad \textbf{eq. S3}$$

$$f_{DH} = \frac{ME_{LYS\_C1} - ME_{DAP\_C7}}{2 \times ME_{OAA\_C1} - ME_{LYS\_C1} - ME_{DAP\_C7}} \qquad \textbf{eq. S4}$$

Equation S3 and S4 rely on different labelling information. For increasing the reliability of our results, flux calculations were performed with both variants.

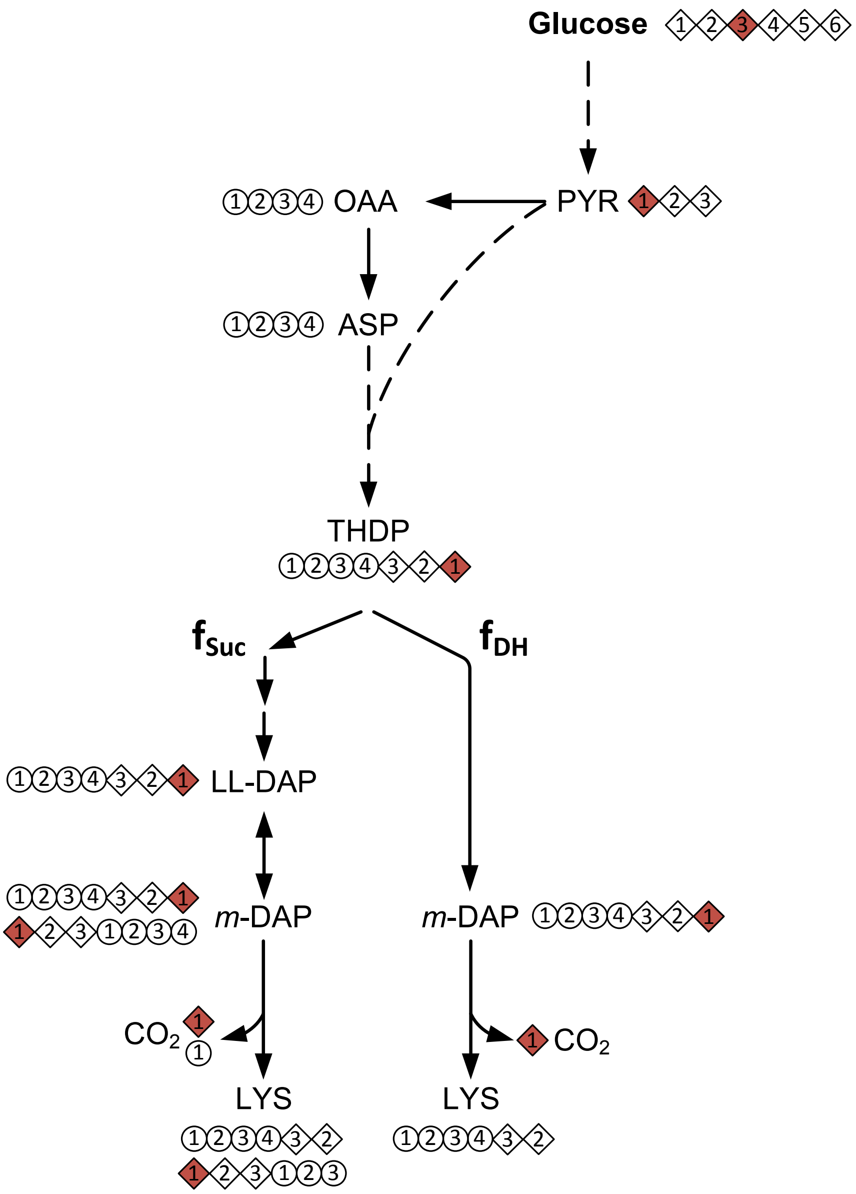

Figure S1: Carbon transition of the succinylase branch (SUC) and the dehydrogenase branch (DH) of the lysine split pathway. The final step of lysine biosynthesis involves decarboxylation of diaminopimelate (DAP) which specifically releases the carbon C_7 of DAP as CO_2. If lysine is formed via the DH branch, the released carbon exclusively originates from carbon C_1 of the lysine building block PYR. However, lysine formation via the SUC branch equally releases carbon C_1 from OAA and PYR due to the presence of a reversible DAP epimerase reaction.

As it is not possible to directly quantify the positional ^{13}C enrichment by a GC-MS based approach, we used a differential method. We thereby took advantage from the specific fragmentation pattern resulting from electron impact ionization during GC-MS measurement [1]. Analysis in this work involved derivatization of the functional groups using MBDSTFA (N-methyl-N-tert-butyldimethylsilyl-trifluoroacetamide). Ionization of the resulting tertbutyl-dimethylsilyl (TBDMS) derivatives by electron impact ionization typically leads to [M-57], [M-85] and [M-159] fragments (Figure S2). The [M-57] fragment thereby contains the complete carbon backbone of the analytes whereas in the [M-85] and the [M-159] fragment, the C_1-carbon is missing. This can be used to differentially quantify the ^{13}C enrichment of the C_1-carbon.

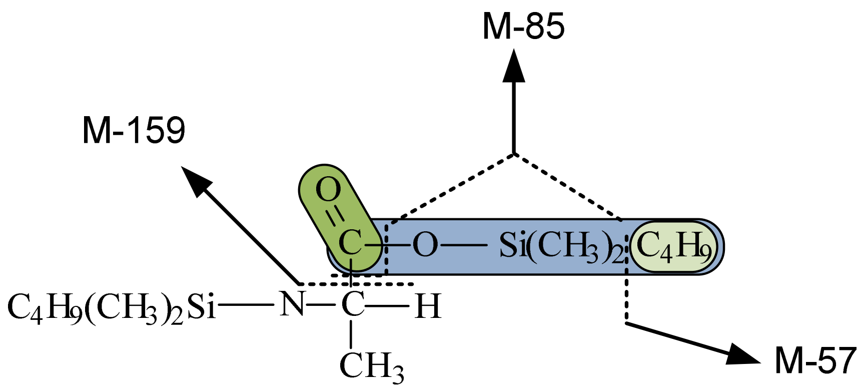

Figure S2: Fragmentation pattern resulting from electron impact ionization of the TBDMS-derivative of the amino acid alanine.

Selected ion monitoring was hence performed for the [M-57] fragments for alanine (ALA, m/z 260), aspartate (ASP, m/z 418), lysine (LYS, m/z 431), and diaminopimelate (DAP, m/z 589), for the [M-85] fragment of alanine (m/z 232), and for the [M-159] fragment of aspartate (m/z 316), and lysine (m/z 329) (Table S1).

Table S1: Mass fragments of TBDMS derivatives of proteinogenic amino acids and of diaminopimelate used for calculation of the lysine split ratio. The given mass refers to the non-labeled M_0 mass isotopomer of the corresponding fragment solely comprising non-labeled C, H, N, O, S, and Si from the analyte itself and the derivatization, respectively.

Analyte	M_0 [m/z]	Fragment	Carbon atoms
Alanine	260	M-57	C_1-C_3
	232	M-85	C_2, C_3
Aspartate	418	M-57	C_1-C_4
	316	M-159	C_2-C_4
Lysine	431	M-57	C_1-C_6
	329	M-159	C_2-C_6
Diaminopimelate	589	M-57	C_1-C_7

After correcting the raw data for the presence of natural isotopes [3], they were used to calculate the molar ^{13}C enrichment of the analyzed sample [4]. The corrected labeling data are given in Table S2 for the labeling experiment with 5 g L^{-1} ammonium sulfate and 50 g L^{-1} ammonium sulfate, respectively.

Table S2: Relative mass isotopomer fraction of alanine (ALA), aspartate (ASP), lysine (LYS) and diaminopimelate (DAP) from hydrolyzed biomass samples of *C. glutamicum* LYS-1 grown in minimal medium with [3-^{13}C] glucose. The medium was supplemented with **(A)** 5 g L^{-1} ammonium sulfate (low ammonium) and **(B)** 50 g L^{-1} ammonium sulfate (high ammonium), respectively. The raw data from GC-MS measurement were corrected for the presence of natural isotopes [3]. M_0 represents the amount of the non-labeled mass isotopomer fraction, M_1 the amount of single-labeld mass isotopomer fraction and corresponding terms refer to higher labeling. In addition, the molar enrichment (ME) [4] is indicated for each fragment. Data for two replicates are given (R1, R2).

	**ALA**				**ASP**				**LYS**				**DAP**	
	**m/z_260**		**m/z_232**		**m/z_418**		**m/z_316**		**m/z_431**		**m/z_329**		**m/z_589**	
**(A)**	**R1**	**R2**	**R1**	**R2**	**R1**	**R2**	**R1**	**R2**	**R1**	**R2**	**R1**	**R2**	**R1**	**R2**
M_0	0.596	0.594	0.854	0.851	0.577	0.576	0.715	0.725	0.475	0.469	0.622	0.620	0.363	0.355
M_1	0.332	0.335	0.136	0.137	0.318	0.324	0.243	0.238	0.366	0.358	0.303	0.304	0.375	0.378
M_2	0.067	0.067	0.010	0.012	0.090	0.086	0.034	0.028	0.133	0.144	0.065	0.065	0.194	0.199
M_3	0.005	0.005			0.015	0.134	0.008	0.009	0.023	0.027	0.009	0.010	0.059	0.058
M_4					0.000	0.001			0.003	0.001	0.001	0.001	0.008	0.010
M_5									0.000	0.000	0.000	0.000	0.000	0.000
M_6									0.000	0.000			0.000	0.000
M_7													0.000	0.000
**ME**	**0.480**	**0.483**	**0.156**	**0.161**	**0.544**	**0.538**	**0.335**	**0.322**	**0.713**	**0.728**	**0.466**	**0.470**	**0.973**	**0.990**
**(B)**														
M_0	0.582	0.583	0.855	0.849	0.559	0.562	0.714	0.711	0.474	0.477	0.615	0.616	0.328	0.334
M_1	0.342	0.341	0.134	0.138	0.333	0.330	0.244	0.244	0.366	0.365	0.305	0.305	0.381	0.382
M_2	0.069	0.070	0.011	0.013	0.094	0.094	0.034	0.037	0.132	0.139	0.068	0.067	0.212	0.214
M_3	0.006	0.006			0.014	0.013	0.008	0.008	0.028	0.016	0.010	0.010	0.067	0.063
M_4					0.000	0.000			0.001	0.003	0.001	0.001	0.011	0.007
M_5									0.000	0.002	0.000	0.000	0.000	0.000
M_6									0.000	0.000			0.000	0.000
M_7													0.000	0.000
**ME**	**0.499**	**0.498**	**0.156**	**0.164**	**0.565**	**0.559**	**0.336**	**0.342**	**0.716**	**0.706**	**0.479**	**0.476**	**1.051**	**1.028**

Subsequently, the labeling information from the different fragments was used to calculate the ^{13}C enrichment of the C_1 carbon of PYR (corresponding to that of alanine), OAA (corresponding to that of aspartate) and of lysine (equations S5 – S7). Additionally, the ME of the C_7 carbon of DAP was quantified from the M-57 fragment of DAP and the M-57 fragment of lysine, respectively (equation S8). This was justified from the fact, that DAP decarboxylase specifically releases the C_7 carbon of DAP while forming lysine (Figure S1).

$$ME_{PYR\_C1} = ME_{ALA260} - ME_{ALA232} \qquad \textbf{eq. S5}$$

$$ME_{OAA\_C1} = ME_{ASP418} - ME_{ASP316} \qquad \textbf{eq. S6}$$

$$ME_{LYS\_C1} = ME_{LYS431} - ME_{LYS329} \qquad \textbf{eq. S7}$$

$$ME_{DAP\_C7} = ME_{DAP589} - ME_{LYS431} \qquad \textbf{eq. S8}$$

The results from calculation of the flux split ratio are given in Table S3.

Table S3: Relative contribution of the dehydrogenase branch to the overall lysine flux which was set to 100 %. The given data refer to flux calculations according to equations 3 and 4, respectively, relying on labeling information from LYS_C1, DAP_C7, and PYR_C1 (eq. 3) and OAA_C1 (eq. 4), respectively. Fluxes were determined individually for the two data sets (Table S2) and used to calculate mean values and standard deviations.

	Low ammonium	High ammonium
$f_{DH\ (eq.\ S3)}$ [%]	6.6 ± 3.2	82.2 ± 4.6
$f_{DH\ (eq.\ S4)}$ [%]	10.2 ± 5.7	81.6 ± 4.9
$f_{DH\ (mean)}$ [%]	8.4 ± 4.3	81.9 ± 3.9

Abbreviations

ALA: alanine; ASP: aspartate; DAP: diaminopimelate; DDH: diaminopimelate dehydrogenase; DH branch: dehydrogenase branch of lysine biosynthesis f_{DH}: relative flux through dehydrogenase branch; f_{Suc}: relative flux through succinylase branch; LYS: lysine; MBDSTFA: N-methyl-N-tert-butyldimethylsilyl-trifluoroacetamide; ME: molar enrichment; OAA: oxaloacetate; PYR: pyruvate, Suc: succinate; TBDMS: tertbutyl-dimethyl-silyl.

References

1. Wittmann C: **Fluxome analysis using GC-MS.** *Microb Cell Fact* 2007, **6:**6.
2. Sonntag K, Eggeling L, De Graaf AA, Sahm H: **Flux partitioning in the split pathway of lysine synthesis in *Corynebacterium glutamicum*. Quantification by ^{13}C- and ^{1}H-NMR spectroscopy.** *Eur J Biochem* 1993, **213:**1325-1331.
3. van Winden WA, Wittmann C, Heinzle E, Heijnen JJ: **Correcting mass isotopomer distributions for naturally occurring isotopes.** *Biotechnol Bioeng* 2002, **80:**477-479.
4. Wittmann C: **Metabolic flux analysis using mass spectrometry.** *Adv Biochem Eng Biotechnol* 2002, **74:**39-64.
