# Supplementary material for: Systems metabolic engineering of Corynebacterium glutamicum for production of the chemical chaperone ectoine
Source: Microb Cell Fact. 2013 Nov 15;12:110. doi: 10.1186/1475-2859-12-110 (PMC4225761; doi:10.1186/1475-2859-12-110)
Supplement: Additional file 2 — Gene_cluster_alignment. [file 1475-2859-12-110-S2.pdf]

```
                                                                  Section 1
                         (1) 1       10        20        30              47
EctSC_codon_optimized    (1) AGCGTATTCTCTGCAACTAGTGCATGTTGGATGCAATGGTTGCAGCG
EctSC_native             (1) AGCGTATTCTCTGCAACTAGTGCATGTTGGATGCAATGGTTGCAGCG
                                                                  Section 2
                        (48) 48          60        70        80          94
EctSC_codon_optimized   (48) CCACTGAGCATCTTGGGAACCTCATGCATGAGCCGCAACACCATCTG
EctSC_native            (48) CCACTGAGCATCTTGGGAACCTCATGCATGAGCCGCAACACCATCTG
                                                                  Section 3
                        (95) 95   100       110       120       130     141
EctSC_codon_optimized   (95) CCCACCGTTGGAATAGCCAACAATAAAGATCCTCTTGATGCCATACG
EctSC_native            (95) CCCACCGTTGGAATAGCCAACAATAAAGATCCTCTTGATGCCATACG
                                                                  Section 4
                       (142) 142     150       160       170            188
EctSC_codon_optimized  (142) TGTTGCCCAAGTGCGTGGCGAGTTTTACAAAGAACCCCACATCATCA
EctSC_native           (142) TGTTGCCCAAGTGCGTGGCGAGTTTTACAAAGAACCCCACATCATCA
                                                                  Section 5
                       (189) 189        200       210       220         235
EctSC_codon_optimized  (189) ATGCCTAAATGGCGGGTATTTTCATCCAAACCCAACCGCGCATCATT
EctSC_native           (189) ATGCCTAAATGGCGGGTATTTTCATCCAAACCCAACCGCGCATCATT
                                                                  Section 6
                       (236) 236           250       260       270     282
EctSC_codon_optimized  (236) CCAATGCTGATCCACCCCATCCGGATAAACCACCATGAACGGCAACG
EctSC_native           (236) CCAATGCTGATCCACCCCATCCGGATAAACCACCATGAACGGCAACG
                                                                  Section 7
                       (283) 283    290       300       310             329
EctSC_codon_optimized  (283) GATCAAAAGTCCTGTTGGTGAAGCTGCGCCCCACAGATCCTGACTGC
EctSC_native           (283) GATCAAAAGTCCTGTTGGTGAAGCTGCGCCCCACAGATCCTGACTGC
                                                                  Section 8
                       (330) 330       340       350       360          376
EctSC_codon_optimized  (330) TGGGAGCCATGAAAATAGATCAGCGCATCCGTGGTGGAACCAAAAGG
EctSC_native           (330) TGGGAGCCATGAAAATAGATCAGCGCATCCGTGGTGGAACCAAAAGG
                                                                  Section 9
                       (377) 377          390       400       410       423
EctSC_codon_optimized  (377) CTCAACAATACGAAACGTTCGCTTTCGGTCCTGATGAAAGAGATGTC
EctSC_native           (377) CTCAACAATACGAAACGTTCGCTTTCGGTCCTGATGAAAGAGATGTC
```

Section 10

(424) 424 430 440 450 460 470
EctSC_codon_optimized (424) CCTGAATCATCATCTAAGTATGCATCTCGGTAAGCTCGACCAGGACA
EctSC_native (424) CCTGAATCATCATCTAAGTATGCATCTCGGTAAGCTCGACCAGGACA

Section 11

(471) 471 480 490 500 517
EctSC_codon_optimized (471) GTGCCACCACAATTTTGGAGGATTACAAGAACATGACCAACATCCGC
EctSC_native (471) GTGCCACCACAATTTTGGAGGATTACAAGAACATGACCAACATCCGC

Section 12

(518) 518 530 540 550 564
EctSC_codon_optimized (518) GTAGCTATCGTGGGCTATCTAGAGTACCTGGGACGCAGCGTCGAAAA
EctSC_native (518) GTAGCTATCGTGGGCTATCTAGAGTACCTGGGACGCAGCGTCGAAAA

Section 13

(565) 565 570 580 590 600 611
EctSC_codon_optimized (565) GTGGCCGTTACCCTGCGAATGTCCACAGGGTAGCTGGTAGTTTGAAA
EctSC_native (565) GTGGCCGTTACCCTGCGAATGTCCACAGGGTAGCTGGTAGTTTGAAA

Section 14

(612) 612 620 630 640 658
EctSC_codon_optimized (612) ATCAACGCCGTTGCCCTTAGGATTCAGTAACTGGCACATTTTGTAAT
EctSC_native (612) ATCAACGCCGTTGCCCTTAGGATTCAGTAACTGGCACATTTTGTAAT

Section 15

(659) 659 670 680 690 705
EctSC_codon_optimized (659) GCGCTAGATCTGTGTGCTCAGTCTTCCAGGCTGCTTATCACAGTGAA
EctSC_native (659) GCGCTAGATCTGTGTGCTCAGTCTTCCAGGCTGCTTATCACAGTGAA

Section 16

(706) 706 720 730 740 752
EctSC_codon_optimized (706) AGCAAAACCAATTCGTGGCTGCGAAAGTCGTAGCCACCACGAAGTCC
EctSC_native (706) AGCAAAACCAATTCGTGGCTGCGAAAGTCGTAGCCACCACGAAGTCC

Section 17

(753) 753 760 770 780 799
EctSC_codon_optimized (753) AGGAGGACATACAATGCCAACCCTGAAGCGCAACTCCATCAACAACC
EctSC_native (753) AGGAGGACATACAATGCCTACCCTAAAAAGGAATTCAATCAACAACC

Section 18

(800) 800 810 820 830 846
EctSC_codon_optimized (800) CAAAGGGCATCGTGCTGTCCTTCCCAACCGTGATGCTGCGTCGCCCA
EctSC_native (800) CCAAAGGCATTGTTTTGAGTTTCCCCACCGTAATGCTCCGTCGCCCA

```
                                                                     Section 19
                          (847) 847          860       870       880          893
EctSC_codon_optimized     (847) ACCGATGGCGACGGCTACAACCTGCACCAGCTCGTGGCACGCTGCCA
EctSC_native              (847) ACCGACGGCGACGGTTACAACCTTCATCAGCTGGTGGCGCGCTGCCA
                                                                     Section 20
                          (894) 894   900       910       920       930       940
EctSC_codon_optimized     (894) GCCACTGGATACCAACTCCGTGTACTGCAACCTGCTGCAGTGCTCCG
EctSC_native              (894) GCCCCTCGATACCAATTCGGTCTACTGCAACCTGCTGCAGTGTTCCG
                                                                     Section 21
                          (941) 941      950       960       970              987
EctSC_codon_optimized     (941) ATTTCGCAGATACCGCAATCGCAGCAGAAAACGCACAGGGCGAACTG
EctSC_native              (941) ATTTCGCTGACACCGCCATCGCCGCAGAGAACGCCCAAGGCGAGCTG
                                                                     Section 22
                          (988) 988         1000      1010      1020         1034
EctSC_codon_optimized     (988) GTGGGCTTCATCTCCGGCTACCGTCCACCATCCCGTCCAGATACCCT
EctSC_native              (988) GTCGGTTTCATCTCGGGTTACCGCCCCCCTTCGCGGCCGGACACGCT
                                                                     Section 23
                         (1035) 1035 1040      1050      1060      1070      1081
EctSC_codon_optimized    (1035) GTTCGTGTGGCAGGTCGCAGTGGATTCCTCCATGCGTGGCCAGGGCC
EctSC_native             (1035) GTTCGTCTGGCAGGTCGCCGTCGACAGTTCGATGCGCGGTCAGGGGC
                                                                     Section 24
                         (1082) 1082   1090      1100      1110              1128
EctSC_codon_optimized    (1082) TGGCACTGCGCATGCTGCTGGCACTGACCGCACGCGTGGCACGCGAA
EctSC_native             (1082) TGGCCCTGCGCATGCTGCTGGCACTGACCGCCCGGGTCGCTCGCGAG
                                                                     Section 25
                         (1129) 1129       1140      1150      1160          1175
EctSC_codon_optimized    (1129) TACGGCGTGCGCTACATGGAAACCACCATCTCCCCAGATAACGGTGC
EctSC_native             (1129) TACGGCGTGCGTTACATGGAAACCACCATCTCGCCGGACAACGGGGC
                                                                     Section 26
                         (1176) 1176          1190      1200      1210       1222
EctSC_codon_optimized    (1176) ATCCCAGGCACTGTTCAAACGCGCATTCGATCGCCTGGATGCAAACT
EctSC_native             (1176) GTCACAGGCGCTGTTCAAGCGGGCCTTCGACCGCCTCGATGCCAACT
                                                                     Section 27
                         (1223) 1223  1230      1240      1250               1269
EctSC_codon_optimized    (1223) GCACCACCCGCACCCTGTTCGCACGCGATACCCACTTCGCAGGCCAG
EctSC_native             (1223) GCACGACGCGCACGCTGTTTGCCCGCGACACGCATTTCGCCGGTCAG
```

```
                                                                    Section 28
                         (1270) 1270      1280      1290      1300           1316
EctSC_codon_optimized (1270) CACGAAGATGAAGTGCTGTACCGTGCAGGCCCATTCACCGTGTCCCA
          EctSC_native (1270) CACGAGGACGAGGTGCTCTACCGCGCCGGCCCGTTCACCGTTTCCCA
                                                                    Section 29
                         (1317) 1317         1330      1340      1350        1363
EctSC_codon_optimized (1317) CCTGGAAGAAGAACTGAAAGAACACGCATAAGAAGTCCAGGAGGACA
          EctSC_native (1317) TCTAGAAGAAGAGCTCAAGGAGCACGCATGAGAAGTCCAGGAGGACA
                                                                    Section 30
                         (1364) 1364  1370      1380      1390      1400     1410
EctSC_codon_optimized (1364) TACAATGAAAACCTTCGAACTGAACGAATCCCGTGTGCGCTCCTACT
          EctSC_native (1364) TACAATGAAAACTTTTGAACTGAATGAATCCAGGGTTCGCAGCTACT
                                                                    Section 31
                         (1411) 1411     1420      1430      1440            1457
EctSC_codon_optimized (1411) GCCGCTCCTTCCCAGTGGTGTTCAAGCAGGCACAGGGTGCAGAACTG
          EctSC_native (1411) GCCGTTCCTTCCCCGTGGTCTTCAAGCAGGCCCAGGGCGCCGAACTG
                                                                    Section 32
                         (1458) 1458        1470      1480      1490         1504
EctSC_codon_optimized (1458) GTGACCCAGGATGGCAAGCGCTACATCGATTTCTTGGCAGGCGCAGG
          EctSC_native (1458) GTCACTCAGGACGGCAAGCGCTACATCGACTTCCTCGCTGGTGCCGG
                                                                    Section 33
                         (1505) 1505 1510      1520      1530      1540      1551
EctSC_codon_optimized (1505) CACCCTGAACTACGGCCACAACCACCCAGTGCTGAAGCAGGCACTGC
          EctSC_native (1505) CACGCTCAACTACGGGCACAACCACCCGGTGCTCAAGCAGGCGCTGC
                                                                    Section 34
                         (1552) 1552    1560      1570      1580             1598
EctSC_codon_optimized (1552) TGGAATACATCGAATCCGATGGCATCACCCACGGCCTGGATATGTAC
          EctSC_native (1552) TCGAGTACATCGAGAGCGACGGCATCACCCACGGCCTGGACATGTAC
                                                                    Section 35
                         (1599) 1599       1610      1620      1630          1645
EctSC_codon_optimized (1599) ACCGAAGCAAAAGAACGCTTCCTGGAAACCTTCAACCGCCTGATCCT
          EctSC_native (1599) ACCGAAGCCAAGGAGCGTTTCCTCGAAACCTTCAACCGGCTGATCCT
                                                                    Section 36
                         (1646) 1646          1660      1670      1680       1692
EctSC_codon_optimized (1646) GGAACCACGCGGTATGGGCGATTACCGCATGCAGTTCACCGGTCCAA
          EctSC_native (1646) CGAGCCGCGCGGCATGGGCGACTACCGCATGCAGTTCACCGGCCCGA
```

Section 37

```
                       (1693) 1693      1700      1710      1720                1739
EctSC_codon_optimized  (1693) CCGGCACCAACGCAGTGGAAGCAGCAATGAAGCTGGCACGCAAGGTG
EctSC_native           (1693) CCGGCACCAACGCGGTCGAGGCGGCGATGAAGCTGGCGCGCAAGGTC
```

Section 38

```
                       (1740) 1740      1750      1760      1770                1786
EctSC_codon_optimized  (1740) ACCGGTCGCAACAACATCATTTCCTTCACCAACGGCTTCCACGGCTG
EctSC_native           (1740) ACCGGGCGCAACAACATCATCAGTTTCACCAACGGCTTCCACGGCTG
```

Section 39

```
                       (1787) 1787      1800      1810      1820                1833
EctSC_codon_optimized  (1787) CTCCATCGGTGCACTGGCAGCAACCGGCAACCAGCACCACCGTGGCG
EctSC_native           (1787) CAGCATTGGCGCGCTGGCCGCCACCGGCAACCAGCATCACCGCGGCG
```

Section 40

```
                       (1834) 1834      1840      1850      1860      1870      1880
EctSC_codon_optimized  (1834) GTTCCGGCATCGGCCTGACCGATGTGTCCCGCATGCCATACGCAAAC
EctSC_native           (1834) GCTCCGGCATCGGCCTCACCGATGTCAGCCGCATGCCGTACGCCAAC
```

Section 41

```
                       (1881) 1881      1890      1900      1910                1927
EctSC_codon_optimized  (1881) TACTTCGGCGATAAGACCAACACCATCGGCATGATGGATAAGCTGCT
EctSC_native           (1881) TATTTCGGCGACAAGACCAACACCATCGGCATGATGGACAAGCTGCT
```

Section 42

```
                       (1928) 1928      1940      1950      1960                1974
EctSC_codon_optimized  (1928) GTCCGATCCATCCTCCGGCATCGATAAGCCAGCAGCAGTGATCGTGG
EctSC_native           (1928) CTCCGACCCGTCCAGCGGGATCGACAAGCCCGCCGCGGTGATCGTCG
```

Section 43

```
                       (1975) 1975      1980      1990      2000      2010      2021
EctSC_codon_optimized  (1975) AAGTGGTGCAGGGCGAAGGCGGTCTGAACACCGCATCCGCAGAATGG
EctSC_native           (1975) AGGTGGTCCAGGGCGAAGGCGGTCTGAACACAGCATCGGCCGAGTGG
```

Section 44

```
                       (2022) 2022      2030      2040      2050                2068
EctSC_codon_optimized  (2022) ATGCGCAAGCTGGAAAAGCTGTGCCGCAAGCACGAAATGCTGCTGAT
EctSC_native           (2022) ATGCGCAAGCTCGAGGAAGCTCTGCCGCAAGCACGAGATGCTGCTGAT
```

Section 45

```
                       (2069) 2069      2080      2090      2100                2115
EctSC_codon_optimized  (2069) CGTGGATGATATCCAGGCAGGCTGCGGTCGTACCGGTACCTTCTTCT
EctSC_native           (2069) CGTCGATGACATCCAGGCCGGCTGCGGCCGCACCGGGACTTTCTTCA
```

```
                                                                  Section 46
                      (2116) 2116          2130      2140      2150       2162
EctSC_codon_optimized (2116) CCTTCGAAGAAATGGGCATCCAGCCAGATATCGTGACCCTGTCCAAG
         EctSC_native (2116) GCTTCGAAGAGATGGGCATCCAGCCGGATATCGTCACGCTGTCCAAG
                                                                  Section 47
                      (2163) 2163   2170      2180      2190            2209
EctSC_codon_optimized (2163) TCCCTGTCCGGCTACGGCCTGCCATTCGCAATGGTGCTGCTGCGCCA
         EctSC_native (2163) TCGCTGTCCGGCTACGGCCTGCCGTTCGCCATGGTGTTGCTGCGCCA
                                                                  Section 48
                      (2210) 2210      2220      2230      2240           2256
EctSC_codon_optimized (2210) AGAACTGGATCAGTGGAAGCCAGGCGAACACAACGGCACCTTCCGTG
         EctSC_native (2210) AGAGCTGGACCAGTGGAAGCCCGGCGAACACAACGGCACCTTCCGCG
                                                                  Section 49
                      (2257) 2257         2270      2280      2290        2303
EctSC_codon_optimized (2257) GCAACAACCACGCATTCGTGACCGCAGCAGCAGCAGTGGAACACTTC
         EctSC_native (2257) GCAACAACCATGCATTCGTCACGGCGGCCGCGGCGGTCGAGCACTTC
                                                                  Section 50
                      (2304) 2304  2310      2320      2330      2340     2350
EctSC_codon_optimized (2304) TGGCAGAACGATGCATTCGCAAACTCCGTGAAGGCAAAGGGCAAGCG
         EctSC_native (2304) TGGCAGAACGACGCGTTCGCCAACAGCGTGAAGGCCAAGGGCAAGCG
                                                                  Section 51
                      (2351) 2351     2360      2370      2380            2397
EctSC_codon_optimized (2351) CATTGCAGATGGCATGCAGCGCATCATCCGTCGCCACGGTCCAGACT
         EctSC_native (2351) CATCGCCGACGGCATGCAGCGCATCATCCGTCGCCACGGCCCGGATT
                                                                  Section 52
                      (2398) 2398        2410      2420      2430         2444
EctSC_codon_optimized (2398) CCCTGTTCCTGAAGGGTCGCGGTATGATGATCGGCATCTCCTGCCCA
         EctSC_native (2398) CGCTGTTCCTCAAGGGCCGCGGGATGATGATCGGCATCAGCTGCCCC
                                                                  Section 53
                      (2445) 2445 2450      2460      2470      2480      2491
EctSC_codon_optimized (2445) GATGGCGAAATCGCAGCAGCCGTGTGTCGCCACGCATTCGAAAACGG
         EctSC_native (2445) GATGGCGAGATTGCCGCCGCAGTGTGCCGCCACGCCTTCGAAAACGG
                                                                  Section 54
                      (2492) 2492    2500      2510      2520             2538
EctSC_codon_optimized (2492) CCTGGTGATCGAAACCTCCGGTGCACACTCCGAAGTGGTGAAGTGCC
         EctSC_native (2492) CCTGGTGATCGAGACCAGCGGCGCCCACAGCGAAGTGGTCAAGTGCC
```

Section 55

(2539) 2539 2550 2560 2570 2585
EctSC_codon_optimized (2539) TGTGCCCACTGATCATCTCCGATGAGCAGATCGATCAGGCACTGTCC
EctSC_native (2539) TCTGCCCGCTGATCATCAGCGATGAGCAGATCGACCAGGCACTTTCC

Section 56

(2586) 2586 2600 2610 2620 2632
EctSC_codon_optimized (2586) ATCCTGGATAAGGCATTCGCAGCAGTGATGTCCGAACAGACCGAAAA
EctSC_native (2586) ATCCTCGACAAGGCCTTTGCCGCCGTGATGAGCGAGCAGACCGAGAA

Section 57

(2633) 2633 2640 2650 2660 2679
EctSC_codon_optimized (2633) CCAGGCATCCTAAGAAGTCCAGGAGGACATACAATGATCGTGCGCAC
EctSC_native (2633) CCAAGCTTCCTAAGAAGTCCAGGAGGACATACAATGATCGTCAGAAC

Section 58

(2680) 2680 2690 2700 2710 2726
EctSC_codon_optimized (2680) CCTGGCAGAATGCGAAAAGACCGATCGCAAGGTGCACTCCCAGACCG
EctSC_native (2680) CCTCGCCGAGTGCGAAAAGACCGACCGCAAGGTCCACAGCCAGACCG

Section 59

(2727) 2727 2740 2750 2760 2773
EctSC_codon_optimized (2727) GCACCTGGGATTCCACCCGCATGCTGCTCAAGGATGATAAGGTGGGC
EctSC_native (2727) GCACCTGGGACAGCACGCGCATGCTGCTCAAGGACGACAAGGTGGGA

Section 60

(2774) 2774 2780 2790 2800 2810 2820
EctSC_codon_optimized (2774) TTCTCCTTCCACATCACCACCATCTACGCAGGCTCCGAAACCCACAT
EctSC_native (2774) TTCTCCTTCCACATCACCACCATCTACGCCGGCAGCGAGACGCACAT

Section 61

(2821) 2821 2830 2840 2850 2867
EctSC_codon_optimized (2821) CCACTACCAGAACCACTTCGAATCCGTCTACTGCATCTCCGGCAACG
EctSC_native (2821) CCACTACCAGAACCACTTCGAGTCGGTGTACTGCATCAGCGGCAATG

Section 62

(2868) 2868 2880 2890 2900 2914
EctSC_codon_optimized (2868) GCGAAATCGAAACCATTGCCGATGGCAAGATCTACAAGATCGAACCA
EctSC_native (2868) GCGAGATCGAAACCATCGCCGACGGCAAGATCTACAAGATCGAGCCG

Section 63

(2915) 2915 2920 2930 2940 2950 2961
EctSC_codon_optimized (2915) GGCACCCTGTACGTGCTGGAAAAGCACGATGAACACCTGTTGCGTGG
EctSC_native (2915) GGCACGCTGTACGTGCTGGAGAAGCATGACGAGCACCTGCTGCGCGG

```
                                                                          Section 64
                      (2962) 2962    2970                2980                2990                    3008
EctSC_codon_optimized (2962) TGGCTCCGAAGATATGAAGCTGGCCTGCGTGTTCAACCCACCACTGA
          EctSC_native (2962) TGGCAGCGAAGACATGAAGCTGGCCTGCGTCTTCAACCCGCCGCTCA
                                                                          Section 65
                      (3009) 3009       3020                3030                3040                  3055
EctSC_codon_optimized (3009) ACGGTCGCGAAGTGCACGATGAATCCGGTGTGTACCCACTGGAAGCA
          EctSC_native (3009) ACGGGCGCGAAGTGCATGACGAAAGCGGCGTCTATCCTCTGGAGGCC
                                                                          Section 66
                      (3056) 3056          3070                3080                3090                3102
EctSC_codon_optimized (3056) GAAACCGTGTAATACCGGAGCTCGATCACGAAGTCCAGGAGGACATA
          EctSC_native (3056) GAAACCGTCTGATACCGGAGCTCGATCACGAAGTCCAGGAGGACATA
                                                                          Section 67
                      (3103) 3103   3110                3120                3130                    3149
EctSC_codon_optimized (3103) CAATGCAGGCAGATCTGTACCCATCCCGTCAAGAAGATCAGCCATCC
          EctSC_native (3103) CAATGCAAGCCGACCTGTATCCCTCGCGCCAGGAAGACCAGCCCAGC
                                                                          Section 68
                      (3150) 3150      3160                3170                3180                  3196
EctSC_codon_optimized (3150) TGGCAAGAACGCCTGGATCCAGTGGTGTACCGCTCCGATCTGGAAAA
          EctSC_native (3150) TGGCAGGAACGCCTGGATCCGGTCGTCTACCGCAGCGACCTGGAGAA
                                                                          Section 69
                      (3197) 3197         3210                3220                3230                3243
EctSC_codon_optimized (3197) CGCACCAATCGCAGCCGAACTGGTGGAACGCTTCGAACGCGACGGCT
          EctSC_native (3197) TGCGCCGATCGCGGCAGAGCTGGTCGAACGCTTCGAACGCGACGGCT
                                                                          Section 70
                      (3244) 3244  3250                3260                3270                3280   3290
EctSC_codon_optimized (3244) ACCTGGTGATCCCAAACCTGTTCTCCGCAGATGAAGTGGCACTGTTT
          EctSC_native (3244) ACCTGGTCATCCCCAATCTGTTCAGCGCCGACGAAGTCGCGCTGTTT
                                                                          Section 71
                      (3291) 3291     3300                3310                3320                  3337
EctSC_codon_optimized (3291) CGCGCAGAACTGGAACGCATGCGCCAGGATCCAGCAGTGGCAGGCTC
          EctSC_native (3291) CGCGCCGAACTCGAGCGCATGCGCCAGGACCCGGCCGTCGCCGGTTC
                                                                          Section 72
                      (3338) 3338        3350                3360                3370                3384
EctSC_codon_optimized (3338) CGGCAAGACCATCAAAGAACCAGATTCCGGTGCAATCCGCTCCGTGT
          EctSC_native (3338) CGGCAAGACCATCAAGGAACCCGACAGCGGTGCGATCCGCTCGGTGT
```

Section 73
(3385) 3385 3390 3400 3410 3420 3431
EctSC_codon_optimized (3385) TCGCAATCCACAAGGATAACGAACTGTTCGCTCGCGTGGCAGCAGAT
EctSC_native (3385) TCGCCATCCACAAGGACAACGAGCTGTTCGCCCGCGTCGCAGCCGAC

Section 74
(3432) 3432 3440 3450 3460 3478
EctSC_codon_optimized (3432) GAACGCACCGCAGGTATCGCACGCTTCATCCTGGGTGGCGATCTGTA
EctSC_native (3432) GAGCGCACCGCCGGCATCGCCCGCTTCATCCTTGGCGGCGACCTGTA

Section 75
(3479) 3479 3490 3500 3510 3525
EctSC_codon_optimized (3479) CGTGCACCAGTCCCGCATGAACTTCAAGCCAGGCTTCACCGGCAAAG
EctSC_native (3479) CGTGCATCAGTCGCGAATGAACTTCAAGCCCGGCTTCACCGGCAAGG

Section 76
(3526) 3526 3540 3550 3560 3572
EctSC_codon_optimized (3526) AATTCTACTGGCACTCCGATTTCGAAACCTGGCACATCGAAGATGGC
EctSC_native (3526) AGTTCTACTGGCACTCGGATTTCGAGACCTGGCACATCGAGGACGGC

Section 77
(3573) 3573 3580 3590 3600 3619
EctSC_codon_optimized (3573) ATGCCACGCATGCGCTGCCTGTCCTGCTCCATCCTGCTGACCGATAA
EctSC_native (3573) ATGCCGCGCATGCGCTGCCTGTCCTGCTCGATCCTCTTGACCGACAA

Section 78
(3620) 3620 3630 3640 3650 3666
EctSC_codon_optimized (3620) CGAACCACACAACGGTCCACTGATGCTGATGCCAGGCTCCCACAAGC
EctSC_native (3620) CGAGCCGCACAACGGCCCGCTGATGCTGATGCCCGGCTCGCACAAGC

Section 79
(3667) 3667 3680 3690 3700 3713
EctSC_codon_optimized (3667) ACTACGTGCGCTGCGTTGGCGCAACCCCAGAAAACCACTACGAAAAG
EctSC_native (3667) ACTACGTGCGCTGCGTCGGAGCCACACCGGAAAATCACTACGAGAAG

Section 80
(3714) 3714 3720 3730 3740 3750 3760
EctSC_codon_optimized (3714) TCCCTGCGCAAGCAAGAAATCGGCATCCCTGATCAGAACTCCCTGTC
EctSC_native (3714) TCCCTGCGCAAGCAGGAGATCGGCATCCCCGACCAGAACAGCCTGAG

Section 81
(3761) 3761 3770 3780 3790 3807
EctSC_codon_optimized (3761) CGAACTGGCATCCCGCTTCGGCATCGATTGCGCAACCGGTCCAGCAG
EctSC_native (3761) CGAGCTGGCCAGCCGCTTCGGCATCGACTGCGCCACCGGCCCCGCCG

```
                                                                Section 82
                         (3808) 3808       3820      3830      3840         3854
EctSC_codon_optimized (3808) GCTCCGTGGTGTTCTTCGATTGCAACACCATGCACGGCTCCAACGGC
         EctSC_native (3808) GCAGCGTGGTGTTCTTCGACTGCAACACCATGCACGGCTCCAACGGC
                                                                Section 83
                         (3855) 3855 3860      3870      3880      3890     3901
EctSC_codon_optimized (3855) AACATCACCCCATCCGCACGCTCCAACCTGTTCTACGTGTACAACCA
         EctSC_native (3855) AACATCACGCCCAGCGCGCGTAGCAATCTGTTCTACGTCTACAACCA
                                                                Section 84
                         (3902) 3902    3910      3920      3930            3948
EctSC_codon_optimized (3902) CGTGGATAACGCAGTGCAGGCACCATTCTGCGAACAGAAGCCACGCC
         EctSC_native (3902) CGTGGATAATGCCGTGCAGGCTCCGTTCTGCGAGCAGAAACCGCGCC
                                                                Section 85
                         (3949) 3949       3960      3970      3980         3995
EctSC_codon_optimized (3949) CAGCATTCGTGGCCGAACGCGAAAACTTCAAGCCTCTGGATATTCGC
         EctSC_native (3949) CGGCCTTTGTCGCCGAACGCGAGAATTTCAAGCCGCTGGACATTCGG
                                                                Section 86
                         (3996) 3996          4010      4020      4030      4042
EctSC_codon_optimized (3996) CCACAGCAGTACCTGTAATCCAGGATCCATACCTGCTCTCCCCAGAG
         EctSC_native (3996) CCGCAACAGTATCTCTGATCCAGGATCCATACCTGCTCTCCCCAGAG
                                                                Section 87
                         (4043) 4043   4050      4060      4070             4089
EctSC_codon_optimized (4043) AATCTAGAGTACCGATCTGATCGCACGCGACGTCTAATTTAGCTCGA
         EctSC_native (4043) AATCTAGAGTACCGATCTGATCGCACGCGACGTCTAATTTAGCTCGA
                                                                Section 88
                         (4090) 4090      4100      4110      4120          4136
EctSC_codon_optimized (4090) GGGGCAAGGAAACAGTGTGGTTTCCTTGCCTCTTTTAGCCTTTTCAG
         EctSC_native (4090) GGGGCAAGGAAACAGTGTGGTTTCCTTGCCTCTTTTAGCCTTTTCAG
                                                                Section 89
                         (4137) 4137         4150      4160      4170       4183
EctSC_codon_optimized (4137) AGGGTGTCTTCGCTGGACCAAGAGGAAACCAGACAGGCGTGACAAAA
         EctSC_native (4137) AGGGTGTCTTCGCTGGACCAAGAGGAAACCAGACAGGCGTGACAAAA
                                                                Section 90
                         (4184) 4184  4190      4200      4210      4220    4230
EctSC_codon_optimized (4184) ATCTGGATTTCCGCCAGGTTTTGGCACGCCTGTCTGGTTTAGGGGAT
         EctSC_native (4184) ATCTGGATTTCCGCCAGGTTTTGGCACGCCTGTCTGGTTTAGGGGAT
```

```
                                                                     Section 91
                    (4231) 4231     4240      4250      4260                4277
EctSC_codon_optimized (4231) GAGAAACCGGACACACGTGCCAAAACTTCGGCTTTTTCGCCAATCTT
          EctSC_native (4231) GAGAAACCGGACACACGTGCCAAAACTTCGGCTTTTTCGCCAATCTT
                                                                     Section 92
                    (4278) 4278        4290      4300      4310           4324
EctSC_codon_optimized (4278) GTCACGCCTGTCTGGTTTGCCTCGGATGAGGTGATTTCATGGCCAAG
          EctSC_native (4278) GTCACGCCTGTCTGGTTTGCCTCGGATGAGGTGATTTCATGGCCAAG
                                                                     Section 93
                    (4325) 4325 4330      4340      4350      4360      4371
EctSC_codon_optimized (4325) ACTTCTAAAAGTTCGACCTCGCAGGATCGCTTCTAAGGGCCTTTAGC
          EctSC_native (4325) ACTTCTAAAAGTTCGACCTCGCAGGATCGCTTCTAAGGGCCTTTAGC
                                                                     Section 94
                    (4372) 4372   4380      4390      4400              4418
EctSC_codon_optimized (4372) GGACCAACCTAGGCCGATACCCATGTGGAAATCTCGACGTCTTAAAT
          EctSC_native (4372) GGACCAACCTAGGCCGATACCCATGTGGAAATCTCGACGTCTTAAAT
                                                                     Section 95
                    (4419) 4419       4430      4440      4450          4465
EctSC_codon_optimized (4419) GGACGATTGGAGCTAAAACCACGAACAGCTGGGATTTTCCACGATAG
          EctSC_native (4419) GGACGATTGGAGCTAAAACCACGAACAGCTGGGATTTTCCACGATAG
                                                                     Section 96
                    (4466) 4466          4480      4490      4500       4512
EctSC_codon_optimized (4466) GATTGGGTCTCGTGGAGATTCGTTGGTTGGAAGGCTTTATCGCGGTC
          EctSC_native (4466) GATTGGGTCTCGTGGAGATTCGTTGGTTGGAAGGCTTTATCGCGGTC
                                                                     Section 97
                    (4513) 4513   4520      4530      4540              4559
EctSC_codon_optimized (4513) GCGGAAGAATTGCACTAGTAATGCTGCGATTCGTTTGGGGATGCCGC
          EctSC_native (4513) GCGGAAGAATTGCACTAGTAATGCTGCGATTCGTTTGGGGATGCCGC
                                                                     Section 98
                    (4560) 4560           4577
EctSC_codon_optimized (4560) AATCGCCGTTGAGTCAGT
          EctSC_native (4560) AATCGCCGTTGAGTCAGT
```
